# Health Research Priority Agenda for Ministry of Health, Kingdom of Saudi Arabia from 2020 to 2025

**DOI:** 10.1007/s44197-022-00061-5

**Published:** 2022-10-04

**Authors:** Athari Alotaibi, Wafaa Saleh, Abdulaziz Abdulbaqi, Maha Alosaimi

**Affiliations:** 1 General Directorate of Research and Studies, Ministry of Health, Riyadh, Kingdom of Saudi Arabia; 2 Health Sector Transformation Program, Riyadh, Kingdom of Saudi Arabia

**Keywords:** Priority setting, Ministry of Health, Kingdom’s Vision 2030, Health system research, Delphi Approach, Research agenda

## Abstract

**Method:**

The current study applied e-Delphi technique via online self-administered questionnaire was distributing to headquarter, and 16 health affairs directorates spanning 75 hospitals and specialized health centers, 24 primary health-care centers, 2 health-care clusters, and 5 medical cities. In addition, community involvement was represented by 26 organizations: 7 universities, 9 scientific health associations, 5 charitable associations, and 5 key Saudi health partner organizations. Research field’s prioritization was performed by ranking weighed mean aggregate score via application of the combined consensus and metrics-based approach. Then the top five research topics were analyzed, verified, refined and classified into specific health research themes.

**Results:**

The study included 2252 participants and attained a 90% response rate. The study deliverables were listed into two research priority domains: health system research priorities (1st agenda) and diseases and health problems priorities (2nd agenda). Overall, the types of the top five research priorities in the first agenda included service delivery (40.9%), health workforce (14.4%), governance and leadership (13.0%) ,preparedness and response to disasters and emergency (10.2%), health information systems (9.3%), access to essential medicines products and vaccines (6.97%), and financing (5.1%). On the other hand, the top five research priority areas in the second agenda were non-communicable diseases (16.9%), child and neonatal health (15.9%), medications (13.6%), women health (10.4%), dental health (10.4%). furthermore, biomedical and radiology technology and devices (5.6%), communicable diseases (3.7%), nutrition (3.2%), trauma and general management (3.2%), innovative approaches (2.4%), emergency management (2.7%), physical therapy and rehabilitation (2.3%), public health (2.3%), holistic approaches to health and wellness, behavior and lifestyle (1.5%), environmental health (0.6%),pilgrims’ health (0.6%), geriatric health (0.3%), and family medicine (0.3%).

**Conclusion:**

Adequate description of the stakeholders and methodology can strengthen legitimacy and credibility and maximize the impact of the priority-setting process. Involvement of policymakers, researchers and funding organizations increases the opportunity of translation into actual research, supports redesigning the research landscape and ensures uptake of results and integration.

**Supplementary Information:**

The online version contains supplementary material available at 10.1007/s44197-022-00061-5.

## Introduction

The Ministry of Health (MoH) and its associated regulatory agencies and advisory bodies are undergoing a transformational reform as part of the Kingdom’s Vision 2030. A significant number of strategic initiatives have been developed to ensure that the Kingdom has a health sector characterized by sustainable funding, appropriate access and continuously improving service quality. The Vision Realization Office (VRO), a function within the MoH, has a mandate to design, operate and monitor these initiatives. The successful and sustainable transformation of Saudi health care requires dedicated research resources and capacity aligned with health system priorities, focus on research into clinical services, health services and population health, an understanding of individual and community health risk, outcome evaluation and appraisal of effectiveness of health-care intervention [1].

Priority setting (PS) is an essential process to align health research activities, resources and capacity across the Kingdom to meet MoH priorities and needs [1]. There is currently no consensus on the definition of research PS, but most definitions refer to a range of activities that involve identifying, prioritizing and achieving a consensus on the research areas or questions of importance to stakeholders. However, guidance is needed on evaluation tools that can be applied to research PS [2, 4]. On the other hand, policymakers require a clear declaration of research findings and implications for practice. The World Health Organization (WHO) emphasizes the use of policy briefs and actionable messages and the conduct of policy dialogs to maintain effective communication of research findings with the most appropriate target audiences [5].

Broad stakeholder involvement (multidisciplinary) is beneficial for the outcomes of a research PS exercise for several reasons. Firstly, it minimizes the chances of research options being overlooked [6]. Secondly, it supports personal accountability, thereby increasing the chances of implementing established priorities. Thirdly, priorities will be adjusted according to the needs of those who will implement and benefit from them. Hence, the overall credibility of the exercise and its impact on health will be improved [7]. Lastly, broad stakeholder participation may prevent unnecessary duplication of efforts and waste of resources [8].

## Research Objective

This study aims to identify, empower and support financing important research projects that can respond to the health needs of the country.

## Literature Review

PS plays an important role in health-care systems, as it guides investments in health care and health research and respects resource constraints [9]. The public health sector in KSA is led and represented by the MoH. The MoH has concentrated on the National Vision 2030 to focus on a number of objectives to enhance the quality of medical care. These objectives concentrate on health-care access, value-based health care and management of road traffic accidents (RTA) and public health [1]. Hence, MoH needs to set research priorities to meet national and international health needs, as well as funding research domains that match the priority agenda.

In addition, the research priority agenda is targeting researchers who solve health problems; therefore, it is very important for MoH to identify and declare their priorities. PS objectives must consider the following criteria: social justice, equitable allocation, efficiency and burden of disease [10]. The WHO analyzed data in five developing countries in the Eastern Mediterranean Region and showed that the explanation behind the wide variation in the national research PS is accounted for by the fragmented and poorly coordinated status of the health research system [11].

The assessment of impact and effectiveness of health research PS necessitates the existence of published information on the implementation or evaluation of these researches [12]. As the research funding is controlled by the interests of research founders, the funded health research does not always serve the interests of health policy and strengthening local health systems [9]. Furthermore, there is a need to make sure that research funds are allocated to high-quality research projects. Research enhances the efficacy of public services and policy and promotes quality of life and health. Moreover, it can give important predictive information about disease risk factors and trends, outcomes of treatment or public health interventions, functional abilities, patterns of care and health-care costs and use [13]. Without national priorities for health research, countries cannot guide research expenditure, promote science, technology and innovation in health, build research capacity or negotiate with partners for targeted funding and long-term efforts [14].

No one method is consistently used in PS. Yoshida (2016) identified 165 studies that set health research priorities, noting that only 60% used a defined method. The Child Health and Nutrition Research Initiative Approach (CHNRI) was the most common (26%), followed by the Delphi method (24%). Of the 40% of studies that did not use a defined method, combinations of expert panel interviews, focus group discussions, literature reviews and questionnaires were listed as alternatives [15].

The narrow scope of scientific research in the Arab region can be linked to the absence of a clear research strategy. Additionally, the focus of conducting research on individuals rather than institutions plays a major role in this narrowing. Another main reason for the scarcity of scientific research is the lack of an adequate budget [16]. Compared to developed countries, Saudi Arabia (KSA) does not have as many researchers and funding as developed countries. Saudi Arabia is trying to increase the level of cooperation over all governmental and non-governmental institutions as a long-term goal due to the growing public expectations and the necessity of public participation [16]. KSA spent 0.3% of the gross domestic product on research, which is a low percentage compared to other countries. By 2015, the government intends to increase research funding to 2.1% of the gross domestic product. According to Alshayea 2013, medical research received 36.8% of total research fund [16].

### Burden of Disease Approach

Global burden of disease (GBD) can be used at the national level to assess current and upcoming health challenges by collecting data that helps to measure the magnitude of disease burden. In the 1990s, the GBD concept was developed to describe death and loss of health due to disease, injury and risk factors for all regions of the world [17], and is defined as the burden that a particular disease process has in a particular area, measured by cost, morbidity and mortality [18]. There are a variety of measures to assess health dimensions such as cause of deaths, health expenditure, years of life lost (YLLs), years lived with disability (YLDs), disability-adjusted life-years (DALYs) and the Healthcare Access and Quality (HAQ) Index [19].

Although the burden of disease approach relates research to burden of disease and determinants, cost-effectiveness and financial flows, it requires the existence of sophisticated health information systems and high levels of statistical expertise [14].

Using burden of disease data has drawbacks, in particular uncertainty of estimating the real situation; it is based on historical data, which is heterogeneous and can be inconsistent and non-comprehensive. Furthermore, we cannot see the whole picture of the health status (temporal trends in mortality, incidence and prevalence). According to the study completed by Stefanos Tyrovolas and his colleagues 2020 in Saudi Arabia, the issues with burden of disease measures were mainly related to the sources of data, estimation uncertainty, lags in data availability, variation in coding practices and limitations of existing analytical tools [19].

The top causes of deaths in Saudi Arabia are cardiovascular diseases, neoplasms, diabetes and kidney diseases, maternal and neonatal disorders, respiratory infections and tuberculosis, and nutritional deficiencies. The ten most frequent causes of DALYs are ordered as: cardiovascular diseases, musculoskeletal disorders, neoplasms, neurological disorders, transport injuries, mental disorders, diabetes and kidney diseases, other non-communicable diseases, unintentional injuries, and maternal and neonatal disorders. Moreover, major risk factors for YLDs were identified as high BMI, high fasting plasma glucose concentration, drug use, low physical activity and dietary risks [19].

### Delphi Approach

The Delphi technique is a structured process, which uses a series of questionnaires (known as “rounds”) to gather information. Many studies in the literature used the Delphi technique due to its flexibility. The Delphi technique is useful, particularly for difficult topics that can be influenced by subjective judgments on a collective basis [20]. Moreover, the e-Delphi technique does not require face-to-face meetings and, therefore, is relatively free of social pressure and dominance of individuals or groups and is inexpensive [21]. Delphi can be applied to prioritize health research by engaging large numbers of participants through online surveys "the wisdom of crowds” [22].

Delphi can be applied to prioritize health research by engaging large numbers of participants through online surveys “the wisdom of crowds” [22].

## Methods

The study team of the General Directorate of Research and Studies (GDRS) developed and pilot tested a self-administered questionnaire in both Arabic and English versions. The GDRS team was led by the General Director and guided by the MoH Advisory Authority. They initiated, developed, carried out and reported the PS process. The PS team included a public health consultant and a specialist, in collaboration with highly qualified managers of health research departments in the involved MoH health affairs directorates and medical cities. Additionally, a group of qualified cooperators helped in the facilitation of the online questionnaire fulfillment as well as data validation and verification.

Research priorities may evolve due to changes in the health system or social and political contexts, and therefore an estimated time frame for its validity may be appropriate. [3] The current PS cycle is scheduled to take place over a 5-year period. Furthermore, it can be extended for a long time.

### Ethical Approval and Consent to Participate

Consent to participate was obtained and the purpose of the study and its significance were provided to all participants in a text format, and the questionnaire linked informed consent form was available for participants before conducting the survey. The study was approved by Central Institutional Review Board (IRB), committee of The General Directorate of Research and Studies at Ministry of Health, Kingdom of Saudi Arabia. The reference number is central IRB log No.2019-0016M.

## Research Project Design and Methodology

Many different approaches to health research prioritization exist, but there is no agreement on what might constitute the gold standard or best practice. Moreover, attempting to produce one best practice is in fact not appropriate, as the optimal approach varies according to the exercise [3]. Designing the right PS exercise requires balancing the achievement of target goals as a measure of public health benefit, versus available resources, time and funding [22].

Therefore, the study team decided to consider the e-Delphi technique for the online survey of the current research PS cycle and to apply the General Directorate of Research and Studies (GDRS)-developed, pilot-tested and self-administered questionnaire in both Arabic and English versions. Stakeholders’ engagement in PS research can help to: (1) ensure that research funding addresses the existing gaps to inform decision-making; (2) facilitate the sharing of responsibility and accountability in the implementation of the research agenda; (3) improve research relevance and legitimacy; and (4) lastly realize better health outcomes [23, 24].

### e-Delphi Technique

The study utilized an e-Delphi method as an interactive forecasting approach, through which a panel of experts answered questionnaires in two rounds and the mean scores defined the agenda [14]. Furthermore, as Delphi is a metric-based approach, databases are used to analyze and rank priorities [22].

Several different methods can be used to decide on priorities, which broadly fall into two groups: consensus-based approaches and metrics-based approaches. The former lead priorities to be decided by group consensus, while the latter involve metrics or an algorithm that results in pooling of individual rankings of research options. Consensus tends to improve the acceptability of the exercise, while individual ranking prevents dominance of a few participants. The Delphi technique is an example of a metrics-based approach [3, 13]. Ranking priorities can be performed per research option, with a set of criteria as guidance [25, 26]. It is essential to differentiate between ranking priority issues and priority research questions. The former could be performed by a broad stakeholder group, while the latter is performed by technical experts [23].

### Study Design

The most widely reported method used to identify priorities in previous studies is expert consultation [25]. These ranged from meetings or surveys that collect expert opinion, to more systematic methods that combine a review of the literature, inclusive Delphi surveys of stakeholders and a recognized method for identifying the priorities against weighted criteria [27]. Although objective approaches to health research prioritization that are solely based on burden of disease data or cost-effective analyses exist, most literature on health research PS that was found, as well as the experts that were consulted considered stakeholder involvement to be a fundamental part of the process [28]. Ideally, PS should involve a broad representation of stakeholders, utilize objective and clearly defined criteria for generating priorities and be evaluated [4].

The current study was conducted throughout 2019–2020. It applied the e-Delphi technique via addressing the burden of disease approach’s criteria by its dimensions, which include the magnitude of a health problem, the likelihood of reducing disease burden through controlling its determinants, cost-effectiveness, the present level of knowledge and current resource flows [29]. The study team applied the combined consensus-based and metrics-based approach according to Viergever et al. (2010), as they noted that approaches combining consensus with some form of metrics are common [3].

### Study Questionnaire

Data were gathered by completing the attached online questionnaire. The questionnaire was developed according to [1], [30] and [31] and composed of three sections:

- Section A: socio-demographic characteristics focused on age, gender, nationality and region.
- Section B: official information involved: job/position, affiliated institution, job title, level of qualification, professional category and specialty [32], in addition to contribution to research activities and policymaking process [9].
- Section C: research priority topics and options covered, questions about health research topics, research topics regarding the MoH initiatives to realize Vision 2030 [1] and collaborative research topics.

The questionnaire also included specific criteria to focus thinking around research priorities and to ensure that important considerations were not overlooked. The six criteria were further divided into sub-criteria termed ‘items’. The list below contains the original six criteria in bold, followed by ‘items’ used for scoring the selected research topics:

1. Appropriateness: availability of pre-existing data, ethically and culturally acceptable, etc.
2. Relevance: equity focus and community concern/demand, problem size and contribution to national objectives.
3. Feasibility: capacity of the system to support the research, financial and human resources available and cultural/political environment.
4. Impact of research outcome: opportunity to implement, use of research results, link of research to policy decisions and overall reduction of the problem, including cost.
5. Opportunity to strengthen collaboration with partners: presence of capable partners, available infrastructure and resources, possibility of collaboration and greater research outcome with partner involvement [30].
6. Urgency: whether information is not urgently needed, information could be used right away but a delay of some months would be acceptable, or data is very urgently needed for decision-making [31].

From the items under the criteria, we selected a final ten to use to score the answers: eight of these items from the first four criteria and the remaining two items from the fifth and sixth criteria. The selected items allowed different research dimensions to be balanced against one another, depending on the identified values or principles of the exercise [25, 26, 31]. The chosen topics were given appropriate scores for each of the selected ten items and scoring was conducted based on a rating scale of 1–3. The total aggregate score out of 30 for the chosen topic was computed by clicking ‘calculate’ at the bottom of the page (metric-based approach). The criteria can be categorized into one of three dimensions: public health benefit (should we do it?), feasibility (can we do it?) and cost [33].

### Study Questionnaire Development and Evaluation

GDRS have developed a pilot-tested, online self-administered questionnaire in accordance with Boateng et al. 2018, focusing on three phases (item development, scale development, and scale evaluation) [33].

Phase 1: Item Development [33].

There are many analogous methods used for setting health research priorities [31, 34], but adjustments are necessary to be aligned with the context and needs. The developed 19 questions in the questionnaire were aggregated into three groups—socio-demographic factors, official information and options for priority research topics—represented by questions 1–6, 7–16, and 17–19, respectively. The questions were made up of a number of formats, including multiple-choice questions, dichotomous options (yes/no) and open-ended questions.

Second: content validity. We assessed if the measure adequately captured the concept’s full meaning or not, and how accurately an assessment or measurement tool tapped into the various aspects of the specific construct in question. Our developed questionnaire was revised and formulated based on five experts’ views to ensure clarity of the meaning, relevance to study objectives and easy understanding by participants. They were ensured by assessing the questionnaire, recording notes, collecting experts’ opinions, discussion, reaching consensus, approving modifications and generating the amended version. Afterward, the questionnaire was assessed and tested by a sample of target experts, to determine which questions should be included in the survey and which should be not. All the construct questions were assessed, topic-relevant questions were included, and it was insured that the questions were representative of all aspects of the construct and could fully measure the relevant domain. After that, the experts were agreed on the assessment tool and the Delphi method was used to come to a consensus on which questions were a reflection of the construct we wanted to measure. The target experts were specialized in research management, health policy implementation, public health and health-care quality.

Expert judgment was completed, and the final version was modified based on focus group discussions’ feedback and on the expert consensus on what items will be accepted, rejected or modified.

Evaluation by the target population was complete through interviews to establish if the items of the assessment tool were appropriate to the topic and a good measure of the topic domains.

The “five experts” were experts in the general directorates of quality, planning, statistics, research and all of the experts working at Assistant Deputyship for Planning and Organizational Excellence at Ministry of Health, KSA.

The target experts were selected based on their position as decision maker and their experience as health researcher and care provider. The majority of them were decision makers, researchers and health-care providers.

Phase 2: Scale Development

Third, tool pre-testing. The study team shared the draft questionnaire with 50 participants over two rounds, to validate the pre-tested questions according to their feedback. Following this, we conducted interviews to further refine and assess item interpretation and finalize the survey tool.

Fourth, survey administration and sample size determination. The sample size was calculated through software. A large sample size (2500 participants) was selected to cover a wide range of specialists, and the study included both quantitative and qualitative data.

Fifth and sixth: items’ reduction and extraction.

The tool was reviewed to identify items that were not or were the least related to the study domain for deletion or modification, subsequently unnecessary variables were deleted.

## Phase 3 Scale Evaluation

Test–retest reliability or simply stability testing was computed through correlation [35], which indicates the degree to which values were consistent through repeated testing. The most direct way of estimating reliability is to manage the test two times with an identical set of themes and then correlate the two measurements at each time point, with the correlation coefficient (r) between the two sets of values designating the degree of reliability [35]. The correlation magnitude for the administered study questionnaire on two occasions separated by 2 weeks was 0.95.

Content validity test: Content validity is an assessment of how fitting the factors being measured are according to a panel of assessors with good subject matter knowledge. It speaks of how precisely a measurement tool taps into the various features of the particular construct in question [35]. There is no correlation coefficient, as this method of testing is a logical method rather than an empirical one, due to dependence on the relevance of the test task with the content of the construct [35]. The developed questionnaire aimed to identify health research priorities for the concerned populations using criteria that allowed different research dimensions to be balanced against one another. The content validity was evaluated as a subjective decision and was highly rated (98%) by a panel of relevant MoH experts.

**Inclusion criteria and strategy for stakeholder’s involvement**

**Fig. 1:**
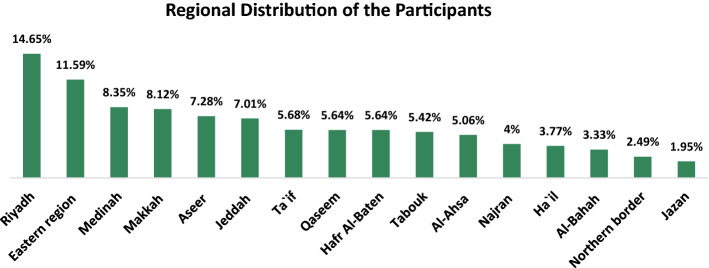
Distribution of study participants amongst ministry of health affairs’ general directorates and directorates

The intended participants for the survey were defined as MoH leaders, along with the full range of MoH health-care professionals' specialties and subspecialties. In addition to the relevant community organization, representatives are as follows:

1. MoH Main Campus: headquarters policy and decision makers at all levels (Main Campus Leader population based). We mean by MoH main campus (headquarter), population-based technique, inviting all general directors and those in higher positions in the hierarchical structure of MOH. MoH high level leaders include headquarters leaders —85 participants and regional decision makers—49 participants.
2. MoH Health Affairs General Directorates and Directorates: (via multistage stratified sampling technique) and recruitment through the selected 16 health affairs directorates (Fig. 1), hospitals, specialized health centers, two health-care clusters and primary health-care centers.
3. The study clusters are composed of the highest 14 health affair regions with regard to the overall physician percentages (3% and more). Every 3 of the other remaining (14 out of 16 regions) 6 regions (physician percentages < 3%) are merged to form two strata, each of them represented by 5% of MoH total physicians. Northern borders and Hafr Al-Baten represented the merged two categories as they have the highest physician density within their clusters.
4. Strategy for Surveying and Sampling Technique:
5. The survey team decided to consider "Major City-based sample” as a comprehensive approach rather than “Hospital-based sample”.
6. The MoH regional sample was estimated for professional categories and regions through the online sample size calculator, which is accessible via the following website: (www.surveysystem.com, https://www.checkmarket.com/sample-size-calculator/) [36]. Due to a wide range of specialties for the physicians and allied health professionals, the estimated samples were multiplied by 300% and 150%, respectively, to cover the varied strata [37]. The data source was the MoH, KSA Statistical Year Book 1438 H [37]. The estimated sample included 75 hospitals and specialized health centers, 24 primary health-care centers, 2 health-care clusters, and 5 medical cities.

## Health and Community Participation

We searched for the online registered organizations, contacted with and invited them to participate as one or more participant/organization), including the seven Saudi universities which have signed in advance a collaborative agreement with GDRS.

The survey steering committee assigned coordinators to communicate with the selected governmental and non-governmental community entities’ representatives [39] and invited them to fulfill the survey questionnaires. The sample was planned to include:

- Registered health scientific associations (No. 40); data source is Saudi Commission for Health Specialties [30]. It was preferable for the questionnaire to be completed by a health-care professional.
- Key Saudi health/partner organizations (No. 15).
- Registered charitable associations (No. 52); data source is MoH Community Participation Program.
- The seven cooperative Saudi universities which assigned academic memorandums of understanding to GDRS were selected. The current survey included; King Saud University, Umm al-Qura University, Riyadh Elm University, Alfaisal University, Prince Sattam bin Abdulaziz University, King Abdulaziz University and Princess Noura University.

### Data Collection

- The data collection process was initiated by sending circular letters by GDRS to invite the enrolled Health Affairs' general directors and directors and medical cities' executive directors to participate in the survey. Other participants were contacted via email.
- The total calculated sample was 2500, while 2252 participants (90% response rate) completed the survey tool.

#### Data Management and Analysis

The study variables were summarized in terms of frequency distribution and by computing quantitative measures, using descriptive statistics (mean aggregate score). Bivariate analysis was performed to test the association between variables (Pearson correlation) using the software program Stata version 15, and qualitative analysis was conducted as appropriate (consensus %) (3.14). Research domains were prioritized by ranking the weighted mean aggregate score, which was computed by multiplying consensus % by mean aggregate score of the selected topic/issue. All topics of the top five domains were included in PS agendas, whereas the lower-order fields were excluded. The methods used to decide between priorities fall broadly into two groups: consensus-based approaches and metrics-based approaches. Consensus-based methods tend toward improving the acceptability of the exercise, while a scoring system dampens down the dominance of the minority of stakeholders [22].

Moreover, collating and categorizing priorities was achieved by application of taxonomy and other frameworks that were used to organize, summarize and aggregate research topics/questions. Furthermore, project team revisions were made according to the scope, adding clarity, definition, avoiding duplication and ensuring validation and verification.

## Results

The study included 2252 participants with a 90% response rate; the majority (68%) were Saudi natives. Their ages varied from 25 to > 60 years, with the majority (76%) aged between 25 and 45 years. Two-thirds of respondents were males and the study achieved balanced regional participation. Inclusiveness was attained in terms of participants representing a full range of health specialties and subspecialties (46.5% physicians, 39% health specialists, 10% pharmacists and 3.6% dentists and related domains 1%) (Table 1).

**Table 1:** Socio-demographic characteristics

Socio-demographic characteristics	Total	
	*n*	**%**
Age	2252	100
25 to < 35 = 1	933	41.43
35 to < 45 = 2	769	34.15
45–60 = 3	496	22.02
More than 60 years, = 4	54	2.4
Gender	2252	100
Male = 1	1,513	67.18
Female = 2	739	32.82
Nationality	2,252	100
Saudi = 1	1,540	68.38
Non-Saudi = 2	712	31.62
Level of education	2,252	100
1 = bachelor’s	1,011	44.89
2 = master’s	447	19.85
3 = medical fellowship	340	15.1
4 = doctoral	411	18.25
5 = others	43	1.91
Stakeholders’ professional classification	2252	99.99
1 = consultant	545	24.2
2 = senior specialist	304	13.5
3 = specialist	1,107	49.16
4 = other, please specify	296	13.14
Professional specialty	2252	100
1 = doctor	1,048	46.54
2 = dentist	81	3.6
3 = pharmacist	219	9.72
4 = health specialist	877	38.94
5 = one of the general specialties related to the of health field	27	1.2
The participants who contributed to scientific research	2252	100
1 = yes, they contributed	1,004	44.58
2 = no, they did not contribute	1,248	55.42

The Kingdom was represented by 16 regions (health affairs directorates) according to Saudi MoH (STATISTICAL YEARBOOK 2018) [31] and their contribution was proportional to health-care providers’ density, as (30%) was achieved by Riyadh, Makkah and Jeddah, whereas only 9% of the total sample belonged to Jazan, Najran and Al-Bahah. Broad stakeholder involvement was achieved as the study covered a wide spectrum of levels of qualification (from bachelor’s degrees [45%] to doctoral degrees [18%] and professional levels (specialist [49%] to consultant [24%]) (Table 1). This mix of specialty from a number of disciplines was included in the study to obtain a fully comprehensive model.

The clear majority of participants (98%) belonged to the MoH, of which 134 were leaders (85 headquarters policy makers and 49 regional decision makers), while the rest were made up of stakeholders from 16 Health Affairs Directorates (Fig. 1) spanning 75 hospitals and specialized health centers, 24 primary health-care centers, 2 health-care clusters and 5 medical cities. Community involvement was represented by 26 organizations: 7 universities, 9 scientific health associations, 5 charitable associations and 5 key Saudi health partner organizations. Nearly half of the stakeholders (45%) contributed to scientific research, while around a quarter (24%) had previously published work. In addition, only 6% had a direct influence in health policymaking.

All international and national collaborative research themes (Agenda 3), along with the vast majority of health system (Agenda 1) and epidemiological research topics and issues were selected by MoH Headquarter leaders. Regional stakeholders focused mainly on clinical research and to a lesser extent on epidemiological domain (Agenda 2).

The study clarified that there was no correlation between contribution to scientific research, publications and the other studied variables such as regions, age, gender and nationality as the correlation coefficient was *r* ~ 0.

It is worth noting that there is clear relationship between stakeholders' job/position and research options. MOH headquarters’ leaders focused on domains relevant to health system, public health and national programs, as well as collaborative research themes. Indeed, they addressed the vital role of health research in transforming, modernizing and managing the health-care system.

On the other hand, leaders and health-care providers at the regional level were more interested in research themes concerned with diseases, health care and hospital administration and in particular workforce development.

**Fig. 2:**
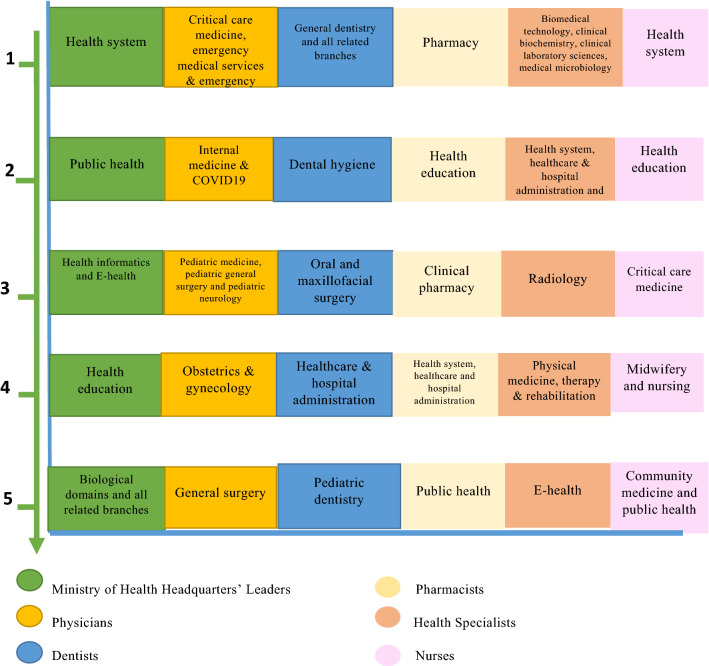
The Top five research priority themes for MoH headquarters’ leaders, all health categories and other Saudi health organizations included in research priority agendas

The project team classified the participants into six categories; for each group, the top five priority fields were ranked and identified. The applied parallel approach kept track for each category and prevented dominance of data belonging to one professional group over the others. The ranking process considered the mixed method approach and combined both consensus- and metric-based methods. According to weighted average overall score rating, the research issues of the top five domains were included in the PS agendas, while the lower-ranked fields were excluded as presented in Fig. 2. The second step included defining the top five priority themes for each track, all research topics and questions of the top five ranked domains, as well as research topics related to VRO office’s initiatives to realize Vision 2030, and collaborative research. All topics options were pooled together, validated, verified, tallied, summarized, refined and classified into themes. The study deliverables were listed into three research priority agendas:

1. Health System Research Priority Agenda included service delivery (40.9%), health workforce (14.4%), governance and leadership (13.0%), preparedness and response to disasters and emergency (10.2%), health information systems (9.3%), access to essential medicines products and vaccines (7.0%) and financing (5.1%).
2. diseases, health problems, public health and medical care agenda; the top research priority areas were non-communicable diseases (16.9%), child and neonatal health (15.9%), medications (13.6%), women health (10.4%), dental health (10.4%). Furthermore, biomedical and radiology technology and devices (5.6%), communicable diseases (3.7%), nutrition (3.2%), trauma and general management (3.2%), innovative approaches (2.4%), emergency management (2.7%), physical therapy and rehabilitation (2.3%), public health (2.3%), holistic approaches to health and wellness**,** behavior and lifestyle (1.5%), environmental health (0.6%), pilgrims’ health (0.6%), geriatric health (0.3%) and family medicine (0.3%).
3. National and International Collaborative Research Themes: major research areas impacted by COVID-19, public health, health-care access, medical care and universal health coverage, value-based health care, health system financing and economics, health information and communication technology, health system governance, health workforce development and health system preparedness and response to emergency. The priorities listed in each theme, area and field were the highly ranked issues and topic options by stockholders based on both consensus- and metric-based evaluation. Within each area, other topics were not included as a priority; for example in Agenda 2: diabetes mellitus (DM) and bronchial asthma predominated among endocrine and respiratory diseases, respectively. In addition, as regards dermatological diseases, only the association between vitamin D deficiency and skin diseases was selected as a priority topic, and head and neck radiology as a health specialty focused on the most common tools for diagnosis of thyroid gland diseases.
4. Moreover, many other fields in each specialty were not included; for example, ethics, medical records, forensic medicine, speech and audiology and sustainable development goals (SDGs) and so on.

## Discussion

**Fig. 3:**
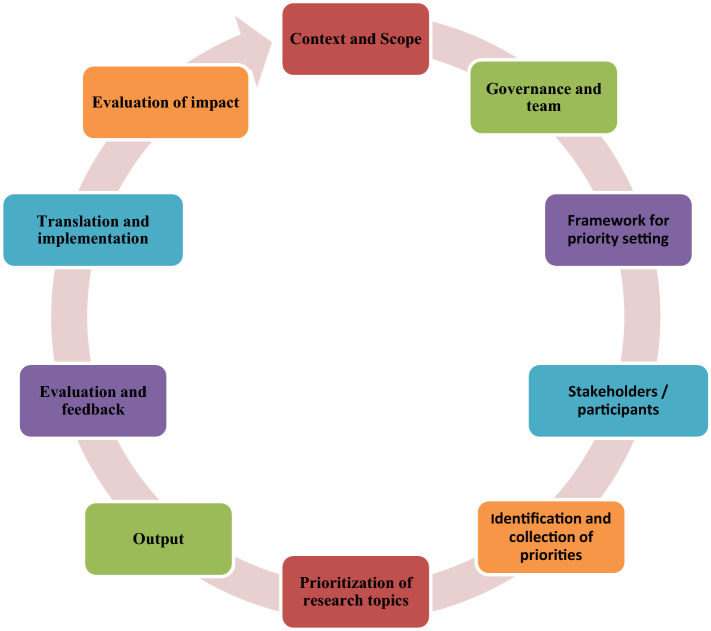
The reporting guideline for health research priority setting

Health research PS should not be a one-time exercise; it is a complete cycle. Comprehensive reporting and improved transparency in research PS study may strengthen the acceptance and implementation of the priorities identified and maximize impact. A reporting checklist for research PS (REPRISE) may facilitate more consistent and comprehensive reporting and enable researchers and end users to better understand the PS processes as shown in Fig. 3 [38].

However, reviews of published research PS exercises have consistently demonstrated a lack of transparency due to suboptimal reporting [23]. Inadequate descriptions of the stakeholders and the methods used make it difficult to assess the validity of research priorities identified and limits the ability to aggregate, analyze or compare the previously established PS exercises [23]. Guidelines are useful for assessing whether the PS process achieves key constructs relevant to the planning, deciding on priorities and post-PS work. Ultimately, the implementation of an effective research PS process will facilitate the allocation of resources to research priorities that have the greatest impact on policy or practice [2]. WHO developed “A systematic approach for undertaking a research priority-setting exercise: guidance”. The document lays forth the methodology to direct the plan, implement, publish, and evaluate (PIPE) phases of the research priority-setting process. WHO assistance can be applied at country level to develop a national research plan or at global or regional scope to coordinate a roadmap for combating a particular disease. Furthermore, the guidance is designed to be adaptable and relevant for use in many different settings and contexts [22].

In terms of context and scope, the study represents MoH headquarters and regional organizations at different levels of care all over Saudi Arabia, under the national umbrella of health and community associations, as representatives of key Saudi health partner organizations and five charitable associations, which represent the civil society. One of the Ministry of Health’s initiatives is Community Empowerment Initiative. It aims to empower community members by giving them a leadership role to formulate the health problems they face, define priorities and make decisions [39].

The study included a full range of MoH health-care professional categories, including headquarters policy and decision makers, directors, managers, multilevel health-care provider specialties and health-related domains.

Along with health community involvement, broad stakeholder participation is beneficial to the impact of a research PS exercise for several reasons: it minimizes the chances of research topics/issues being overlooked; fosters ownership, thus increasing the chances of implementation of the priorities. Lastly, it enables priorities to match the societal and policy needs. Moreover, it may prevent unnecessary duplication of prioritization efforts and hence avoid wasting of resources [21].

According to the Council of Health Research for Development (COHRED)’s 2010 paper, there is not ‘one best method’ for PS [14]. There is no consensus on “successful” research priority-setting approach. But PS processes must be fair, legitimate and transparent and involve broad stakeholders [28].

Each research PS exercise is distinct and specifically designed for the situation. PS team should use the resources to design a PS method that matches their context. [28] Given the various health-care contexts, people, environments, and resource availability in which the priority setting is performed, a wide range of methodologies are applied to prioritize research [4].

Moreover, there is no consensus on the definition of research PS, but most definitions refer to a range of activities that involve identifying, prioritizing and achieving consensus on the research areas/questions of importance to stakeholders [3, 4].

As there is no single agreed upon methodology for the application of the Delphi technique, it allows flexibility in the development of questions and criteria [22].

e-Delphi technique is a systematic, interactive forecasting method, which relies on a panel of experts. Delphi belongs to PS foresight techniques that focus on future health and development [14, 25].

The study included a full range of MoH health-care professional categories, including headquarters policy and decision makers, directors, managers, multilevel health-care provider specialties and health-related domains. Along with community involvement, a little more than half of the stakeholders participated in scientific research, but just 6% directly influenced health policy, while 24% had prior publications. The term "research PS" is not universally agreed upon, although most definitions describe a variety of actions that entail identifying, prioritizing and reaching agreement on the research subjects and issues that are significant to stakeholders [3, 4].

Therefore, the PS study team decided to consider the e-Delphi technique for the online survey of the current research PS cycle. The successful and sustainable transformation of Saudi health care requires: dedicated research resources and capacity aligned with health system priorities, focus on research into clinical services, health services and population health, an understanding of individual and community health risk, outcome evaluation and appraisal of effectiveness of health-care intervention [1]. So, the study questionnaire allowed enrollment of research topic options through the following three topics: health research topics: risk factors, diseases, problems and health system dimensions. Relevant Vision 2030 initiative research topics: health-care access, value-based health care, management of RTAs and public health, along with national and international collaborative research topics. The target beneficiaries of PS exercise are patients, caregivers and general community, whereas the intended audience are those who implement or fund the deliverables of the exercise [3].

KSA general population are the intended beneficiaries through provision of solutions for the gaps and problems relating to their health. Policymakers leading KSA Vision 2030 are interested in PS, as they recognized the integral role of health research in transforming and modernizing the health-care system and improving the health of the Saudi population. Research and development in terms of the need to centralize national research agenda setting and facilitation via funding, grants, research regulations and safety is one of the essential pillars of the future governance framework of Saudi health system transformation. In addition, funders, researchers, industry professionals or others who have the potential to implement the identified priorities are concerned with the outputs of this work.

Regarding PS governance and study team, according to “The Research and Studies by Law”, issued by the Saudi Ministerial Council Decree [23], GDRS is the structure responsible for proposing the health research priority agenda. Following this, the suggested agenda should be shared with the “MoH Research Priority Adoption Committee” for approval. This was put into practice following the application of both the first and second rounds of the e-Delphi technique. It was guided by the process outlined by COHRED in 2010 [13] and modified to accommodate the national governance context.

Priority setting requires credible leadership to support acceptability and uptake. The leadership can be represented by an executive, advisory, or technical expert group [3].

As research, domains were prioritized by ranking the weighted mean aggregate score, which was computed by multiplying consensus % by mean aggregate score of the selected topic/issue. All topics of the top five domains were included in PS agendas, whereas the lower order fields were excluded. The methods used to decide between priorities fall broadly into two groups: consensus-based approaches and metrics-based approaches. Consensus-based approaches improve the acceptability of the exercise, while the scoring system reduces the dominance of minority of stakeholders [22]. Transparency of the current PS process was considered by applying the appropriate method, involving credible leadership, adequate selection and descriptions of the stakeholders, setting context-relevant dimensions and criteria for scoring, along with using the proper data analysis approach to identify priorities. In addition to applying compliance with the updated comprehensive and optimal PS reporting guidelines as regards planning, deciding on priorities and post-PS work are crucial. Moreover, implementing an effective research PS process will facilitate the allocation of resources to research priorities that have the utmost impact on policy or practice.

Previous research has focused on classification of priorities into themes to facilitate implementation [3]. Moreover, others have addressed the sequence of systematic ordering of the research priority agenda, starting with themes, which include priority areas, while fields include relevant terminology [40]. Therefore, the adopted approach for formulating the delivered research priority agendas was via addressing research themes, and areas as broad titles, which were further classified into subtitles for relevant domains of the research topics/questions according to the literature. Extra ordering was avoided to prevent fragmentation and complexity of the agendas.

The Health System Research Domain (Agenda 1) addresses health system and policy questions that are not disease specific, but concern systemic problems that have repercussions on the performance of the health system as a whole. It addresses a wide range of questions, from health financing, governance and policy, to problems with structuring, planning, management, human resources, service delivery, referral and quality of care in the public and private sector [41]. The current study aimed to address health system challenges for providing potential solutions, consequently improving the utility of the findings in other settings. Health system research by necessity is highly multidisciplinary, with a strong emphasis on social sciences, economics and anthropological investigations [42].

The three outcome agendas conform with the proposed goals of the KSA Health Sector Transformation Strategy which are: firstly, to improve health: increase the length, well-being and quality of life of Saudi citizens, which includes the Vision 2030 goal of increasing the life expectancy of citizens to 80 years by 2030; secondly, to improve health care: by improving the quality and consistency of services and the performance and accountability of health-care organizations and staff to deliver care that is safe, effective, patient-centered, timely and equitable; thirdly, to improve value: by containing costs, improving outcomes, controlling public health-care expenditure and guiding new investment [1].

The study deliverables reflected the need for considering holistic approaches to health and wellness, behavior and lifestyle which were addressed in Agenda 2 item IV. In fact, health systems are already struggling to meet current demands and challenges. As a result, solutions may require more innovative approaches that address the wider determinants of health and require multidisciplinary partnerships. The environmental determinants, which include climate change, disasters and emergencies, interact with and potentially have an impact on the social and economic determinants of health, including an aging population and economic decline in many parts of the world [43].

The health system agenda is categorized according to the WHO framework, which describes health systems in terms of six basic components or ‘building blocks’: (i) service delivery, (ii) health workforce, (iii) health information systems, (iv) access to essential medicines, (v) financing, and (vi) leadership/governance**.** The six building blocks contribute to the strengthening of health systems in different ways. Some wider components, such as leadership/governance and health information systems, provide the basis for the overall policy and regulation of all other health system blocks. Key input components include financing and the health workforce. A third group, the medical products, technologies and service delivery, reflected the direct outputs of the health system [44].

Health system research provides evidence that, when applied, it can make health care affordable, safe, effective, equitable, accessible and patient-centric [45, 46].

Service delivery as a prominent goal of the KSA Health Sector Transformation Strategy was enrolled in Agenda 1, along with other factors, including social determinants of health in Agenda 2. The network of service delivery in any well-functioning health system should have the following key characteristics: comprehensiveness, accessibility, coverage, people-centricity, continuity, quality, coordination, accountability and efficiency [44].

The results aligned with the Framework for action for health workforce development in the Eastern Mediterranean Region that has been developed in response to the health workforce challenges facing the region to increase the recruitment, development, training and retention. The framework aims to guide country and regional action to strengthen the health workforce to ensure access for all people to an adequate, competent, well-balanced, motivated and responsive health workforce [47].

The study outcome reflected the interest in the health information system. It comprises the systems relevant to patient’s electronic medical record, the hospital’s operational management or the system supporting health-care policy decisions. Data should be analyzed to improve patient outcomes, inform research and influence policymaking and decision-making [46]. Health information technology (health IT) involves the exchange of health information in an electronic environment. Widespread use of health IT within the health-care industry will improve the quality of health care, prevent medical errors, reduce health-care costs, increase administrative efficiencies, decrease paperwork and expand access to affordable health care [48, 49]. E-Health has been defined as the use of information and communication technologies (ICT) in health products, services and processes combined with organizational change in health-care systems and new skills [50]. e-Health is an umbrella term that covers a wide range of health and care services delivered through ICTs, such as electronic health records (EHRs), health information systems, remote monitoring and consultation services (e.g., telehealth, telemedicine, telecare) [51]. e-Health has experienced a period of significant growth and maturity in recent years. Such investments are most often seen in the context of achieving health system reform, providing innovative modes of health-care delivery or offering efficient methods of access and exchange of health information [52].

Improving access to health products is a multidimensional challenge that requires comprehensive national policies and strategies. Development of an ‘innovation ecosystem’ is a key approach to ensure that companies can collaborate with researchers and to engage effectively in areas of impact to innovate and develop new products, services, solutions and new business models [53].

Health research funding can be an essential function in achieving universal health coverage, through improved effective service coverage and financial protection. Carefully designed and implemented health financing policies can help to address these issues [54]. Moreover, cost-effectiveness, costs and strategic planning can help guide policy decisions to ensure that money spent on health is allocated in a way that the greatest possible health outcomes are achieved in the most feasible manner [55].

Research related to leadership and governance were core themes in the current study. They are influnce by the health service providers (public and private; for and not for profit; clinical, para-medical and non-clinical health services providers; unions and other professional associations; networks of care or of services) and the citizens as service users (population representatives, patients’ associations, civil society organizations, citizens’ associations protecting the poor, etc.) [56].

The results focused on the need for emergency-related research; all communities are at risk of emergencies and disasters including those associated with infectious disease outbreaks, natural disasters and technological hazards, climate change, unplanned urbanization and antimicrobial resistance. Health emergency and disaster risk management emphasizes assessing, communicating and reducing risks across the continuum of prevention, preparedness, readiness, response and recovery, and building the resilience of communities [57].

The present findings (Agenda 2) are consistent with the health sector transformation strategy in Saudi Arabia in showing that rates of avoidable injury and non-communicable disease remain high by regional and international standards. The Kingdom has made notable progress in improving the health, particularly in areas of child and maternal mortality and the reduction of communicable diseases. There is considerable scope to reduce avoidable mortality and morbidity in both the working and elderly populations, with particular areas of concern including heart disease, stroke, DM, respiratory disease, mental health, RTAs and congenital diseases. There is a need to strengthen the prevention of non-communicable disease and injury, thereby reducing avoidable illness and death. The risk of major outbreaks of communicable disease also remains substantial, especially at Hajj or following natural or man-made disasters [1]. KSA is facing a rising burden of non-communicable diseases and road traffic injuries as a result of rapid changes in behaviors, with a resulting clear need for major intervention to reduce these burdens and to engage other sectors of the government and the community in these efforts [58]. Health-care transformation in Saudi Arabia should include a move toward integrative health and medicine, and to promote a culture of wellness [59].

The study stakeholders ranked chronic diseases—including heart disease, stroke, diabetes and cancer as the most common health problems, which coincides with the results of relevant studies in the USA. However, many of these chronic diseases are preventable, as they are linked to poor diet and lifestyle choices including tobacco use, excessive alcohol consumption, and inadequate physical activity [60].

The study deliverables\addressed the goal of oral health to prevent and control oral and craniofacial diseases, conditions, and injuries, and improve access to preventive services and dental care, as the health of the teeth, mouth, and the surrounding craniofacial structures is central to a person’s overall health and well-being. Oral and craniofacial diseases and conditions include: dental caries, periodontal diseases, cleft lip and palate, oral and facial pain, oral and pharyngeal cancers and xerostomia. There are also social determinants that affect oral health. In general, people with lower levels of education and income, and people from specific racial/ethnic groups, have higher rates of disease. People with disabilities and other health conditions, such as diabetes, are also more likely to have poor oral health [61].

Pharmaceutical products research as a fundamental component of both modern and traditional medicine was conducted. Furthermore, it is essential that such products are safe, effective and of good quality, and are prescribed and used rationally [62].

Agenda 3 displayed collaborative research topics. Truly collaborative practices take considerable time and effort to mature, and to deliver effective results. Therefore, the current agenda is an effort to support successful research collaborations. Healthy collaboration built upon shared goals and interests provides added value to the process and ensures a higher potential to achieve a project’s goals [63].

There is a clear need to adopt a “collaborative research agenda” as complex health problems should be addressed in a comprehensive way. The confronted complexity requires input from multiple expert areas, pooling scientific, technological, human capital resources and the associated data.

Effective collaboration at both micro (among professionals) and macro (participation of public and other stakeholders) levels is important to achieve impact and deliver benefit [63]. Collaborative research topic options were recorded by participants who addressed the holistic and interdisciplinary vision of MoH headquarters leaders.

Several health research priority exercises have been previously conducted. These were context specific and need driven, and so are variable in focus, scope and extent [14]. The Sultanate of Oman summarized health research priorities for the MoH in 2014 into two themes: (1) health system building blocks: service delivery, health workforce, health information system, medical products, vaccines and technology, health funding, leadership and governance; (2) research priorities of diseases and risk factors: chronic non-communicable diseases, congenital anomalies and genetic disorders, RTAs and injuries, age-related diseases, disability, handicap and rehabilitation, health promotion, communicable diseases, malnutrition, eye health, women and child health, school and university students/teenagers, environmental and occupational health. [64] A rigorous PS exercise was undertaken by Ireland’s government to direct their 2018–2023 strategy for health research and development. The delivered agenda included six themes: ICT, health and well-being, food, energy, climate action and sustainability, manufacturing and materials and services and business processes [40].

The planned strategy for priority dissemination and feedback is to declare the established priority via online publication of a peer-reviewed journal article.

For attaining a feasible and sustainable implementation of the established research priorities, the following actions were achieved: (1) The involvement of policymakers, researchers and funding organizations from the beginning will help to increase the opportunity for research priorities to be translated into actual research. (2) Classification of priorities into themes and adapting to global research priorities help to facilitate implementation into actual research. Moreover, supporting written evidence of informed policy briefs and making effective use of health research evidence in policymaking will maximize the impact of the established data.

Plans, strategies or suggestions to evaluate the impact include: assessment of policy briefs submission, policy dialog conduction and range of priority domains translated into actual research. Moreover, research deliverable integration in decision-making, influence of research in solving health problems, funding allocation as well as review of other relevant documents are also included.

## Limitations

Given the evident mismatch between the research interests of patients and researchers, investment into health research may be misdirected to areas of low priority or fail to address important needs of relevant stakeholders. The current health research priority exercise relied mainly on the health-care community in KSA, with limited input from other stakeholders including patients and caregivers. However, people-centeredness, patient satisfaction and related issues were addressed. Another factor is to differentiate between ranking priority issues and priority research questions. The former could be performed by a broad stakeholder group, while the latter by technical experts.

- Many accept burden of disease-based methods as a ‘gold standard’. However, when there is no comprehensive contemporary burden of disease data, using a burden of disease approach may be prohibitive in cost. Instead, considering “developing an updated health information system” as a national priority, and using Delphi method in the interim is the alternative approach according to COHRED [14].
- The current study outputs included integrated themes for health system research that meet the main intent of MoH, whereas burden of disease focuses on diseases, injuries, and risk factors sequelae in terms of DALYs (YLDs and YLLs).

## Conclusion

PS exercise approaches can be tailored to match a specific context and needs. The current study applied the e-Delphi technique via addressing the criteria of the burden of disease. They included the magnitude of a health problem, likelihood of reducing disease burden, cost-effectiveness, present level of knowledge and current resources. The study output included the fundamental three priority agendas: 1. health system research priority themes, which harmonized with the MoH vision and transformation and modernization program; 2. diseases, health problems, public health and medical care themes; 3. national and international collaborative research themes. Adhering to guidelines can facilitate comprehensive reporting of the research PS study, in addition to improved transparency and strengthening the acceptability and implementation of the identified research priorities. Therefore, efforts and funding will be invested in generating evidence that is of importance to all stakeholders.

## Supplementary Information

Below is the link to the electronic supplementary material.

Supplementary file1 (PDF 365 KB)

Supplementary file2 (DOCX 93 KB)

## Data Availability

The authors emphasized that the article,and its supplementary materials comprise the data supporting the findings of this study.

## Abbreviations

KSA: Kingdom of Saudi Arabia
MoH: Ministry of Health
GDRS: General Directorate of Research And Studies
PS: Priority setting
GBD: Global burden of disease
WHO: World Health Organization
RTA: Road traffic accidents
YLLs: Years of life lost
YLDs: Years of life lived with disability
DALYs: Disability-adjusted life-years
HAQ: Health-care access and quality
BMI: Body mass index
COHRED: Council on Health Research for Development
IRB: Central Institutional Review Board

## References

[1] Ministry of Health. Health Sector Transformation Strategy. Ministry of Health and the National Transformation Program. KSA . Report number: V.3,2017. Available from: https://www.moh.gov.sa/en/Ministry/vro/Documents/Healthcare-Transformation-Strategy.pdf [Accessed Mar 2018]. https://www.vision2030.gov.sa/media/0wop2tds/hstp_eng.pdf

[2] Mador RL, Kornas K, Simard A, Haroun V (2016). Using the Nine Common Themes of Good Practice checklist as a tool for evaluating the research priority setting process of a provincial research and program evaluation program. Heal Res Policy Syst..

[3] Viergever RF, Olifson S, Ghaffar A, Terry RF (2010). A checklist for health research priority setting: nine common themes of good practice. Health Res Policy Syst..

[4] Bryant J, Sanson-Fisher R, Walsh J, Stewart J (2014). Health research priority setting in selected high-income countries: a narrative review of methods used and recommendations for future practice. Cost Eff Resour Alloc..

[5] Goyet S, Touch S, Ir P, SamAn S, Fassier T, Frutos R, Tarantola A, Barennes H (2015). Gaps between research and public health priorities in low income countries: evidence from a systematic literature review focused on Cambodia. Implement Sci.

[6] Owens C, Ley A, Aitken P (2008). Do different stakeholder groups share mental health research priorities? A four-arm Delphi study. Heal Expect..

[7] Nuyens Y (2007). Setting priorities for health research: lessons from low-and middle-income countries. Bull World Health Organ.

[8] Conceição C, Leandro A, McCarthy M (2009). National support to public health research: a survey of European ministries. BMC Public Health.

[9] Uneke CJ, Ezeoha AE, Ndukwe CD, Gold Oyibo P, Onwe F, Kaur AB (2013). Research priority setting for health policy and health systems strengthening in Nigeria: The policymakers’ and stakeholders’ perspective and involvement. Pan Afr Med J.

[10] Zulu JM, Michelo C, Msoni C, Hurtig AK, Byskov J, Blystad A (2014). Increased fairness in priority setting processes within the health sector: the case of Kapiri-Mposhi District, Zambia. BMC Health Serv Res.

[11] Fedorowicz Z, Waters E, Tugwell P, Nasser M (2007). Health research priority setting in developing countries of the eastern Mediterranean region: partnering with the Cochrane Collaboration. East Mediterr Health J.

[12] McGregor S, Henderson KJ, Kaldor JM (2014). How are health research priorities set in low and middle income countries? A systematic review of published reports. PLoS ONE.

[13] Institute of Medicine (US) Committee on Health Research and the Privacy of Health Information: The HIPAA Privacy Rule. Beyond the HIPAA Privacy Rule: Enhancing Privacy, Improving Health Through Research. Nass SJ, Levit LA, Gostin LO, editors. Washington (DC): National Academies Press (US); 2009. PMID: 20662116. 20662116

[14] Montorzi G, De Haan S, IJsselmuiden C, Kennedy A, Becerra F, Devlin M. Priority setting for research for health. A management process for countries. SN - 92–9226–039–1.Geneva: COHRED. 2010. Available from: http://www.cohred.org/downloads/Priority_Setting_COHRED_approach_August_2010.pdf [Accessed 6th Apr 2018].

[15] Yoshida S (2016). Approaches, tools and methods used for setting priorities in health research in the 21(st) century. J Glob Health.

[16] Alshayea A (2013). Scientific Research in the Kingdom of Saudi Arabia: Potential for Excellence and Indicators of Underdevelopment. High Educ Stud.

[17] Health P, Bank W. Burden of disease : what is it and why is it important for safer food ? 2004;3–5. Available from: http://www.who.int/foodsafety/foodborne_disease/QandA.pdf. Accessed 17 July 2021.

[18] Donev D, Lijana ZK, Burazeri G. Measuring burden of disease : disability-adjusted life years (DALY). A Handb Teach Res Heal Prof. 2013;393–416.

[19] Tyrovolas S, El Bcheraoui C, Alghnam SA, Alhabib KF, Almadi MAH, Al-Raddadi RM (2020). The burden of disease in Saudi Arabia 1990–2017: results from the Global Burden of Disease Study 2017. Lancet Planet Heal..

[20] The Working Group on Priority Setting (2000). Priority setting for health research: lessons from developing countries. Health Policy Plan.

[21] McDonough S, McKenna H, Keeney S, Hasson F, Ward M, Kelly G, Lagan K, Duffy O. A Delphi Study to Identify Research Priorities for the Therapy Professions in Northern Ireland-Executive Summary Report. HSC.Research and Development www.research.hscni.net 2011. Available from: https://www.publichealth.hscni.net/publications/delphi-study-identify-research-priorities-therapy-professions-northern-ireland [Accessed 6th July 2018].

[22] A systematic approach for undertaking a research priority-setting exercise. Guidance for WHO staff. Geneva: World Health Organization; 2020. Licence: CC BY-NC-SA 3.0 IGO.

[23] Tomlinson M, Chopra M, Hoosain N, Rudan I (2011). A review of selected research priority setting processes at national level in low and middle income countries: towards fair and legitimate priority setting. Heal Res Policy Syst..

[24] Bhaumik S, Rana S, Karimkhani C, Welch V, Armstrong R, Pottie K (2015). Ethics and equity in research priority-setting: stakeholder engagement and the needs of disadvantaged groups. Indian J Med Ethics.

[25] Terry RF, Charles E, Purdy B, Sanford A (2018). An analysis of research priority-setting at the World Health Organization—how mapping to a standard template allows for comparison between research priority-setting approaches. Heal Res Policy Syst..

[26] Ranson MK, Bennett SC (2009). Priority setting and health policy and systems research. Heal Res Policy Syst..

[27] Viergever R, Matsoso MP. Health research prioritization at WHO An overview of methodology and high level analysis of WHO led health research priority setting exercises. 2010; p. 29

[28] Lomas J, Fulop N, Gagnon D, Allen P (2003). On being a good listener: Setting priorities for applied health services research. Milbank Q.

[29] Montorzi G, De Haan S, IJsselmuiden C. Council on Health Research for Development (COHRED). Prior Setting Res Heal A Manag Process Ctries. 2010; p. 9–26 (ISBN 92-9226-039). http://www.cohred.org/downloads/Priority_Setting_COHRED_approach_August_2010.pdf. Accessed 20 June 2021.

[30] de Haan S, Kingamkono R, Tindamanyire N, Mshinda H, Makandi H, Tibazarwa F, et al. Setting research priorities across science, technology, and health sectors: The Tanzania experience. Heal Res Policy Syst. 2015;13:14. 10.1186/s12961-015-0002-2. 10.1186/s12961-015-0002-2 PMC4359761 25890313

[31] Yamamoto T. Designing and Conducting Health Systems Research Projects [Internet]. Vol. 1, Corlien M. Varkevisser Indra Pathmanathan Ann Brownlee KIT. Jointly published by KIT Publishers and the International Development Research Centre (IDRC), in association with the Africa Regional Office (AFRO) of the World Health Organization.; 2003. 567–570 p. Available from: http://archives.who.int/prduc2004/Resource_Mats/Designing_1.pdf [Accessed Mar 2018].

[32] Saudi Commission for Health Specialties. Classification and Registration. Available from: https://www.scfhs.org.sa/en/registration/Pages/default.aspx [Accessed Jan 2019].

[33] Boateng GO, Neilands TB, Frongillo EA, Melgar-Quiñonez HR, Young SL (2018). Best practices for developing and validating scales for health, social, and behavioral research: a primer. Front Public Health.

[34] Rudan I, Gibson JL, Ameratunga S, El Arifeen S, Bhutta ZA, Black M, Black RE, Brown KH, Campbell H, Carneiro I, Chan KY, Chandramohan D, Chopra M, Cousens S, Darmstadt GL, Meeks Gardner J, Hess SY, Hyder AA, Kapiriri L, Kosek M, Lanata CF, Lansang MA, Lawn J, Tomlinson M, Tsai AC, Webster J, Child Health and Nutrition Research Initiative (2008). Setting priorities in global child health research investments: guidelines for implementation of CHNRI method. Croat Med J.

[35] Hajjar ST EL. Statistical analysis: internal-consistency reliability and construct validity Said Taan EL Hajjar Ahlia University. Int J Quant Qual Res Methods [Internet]. 2018;6(1):27–38. Available from: www.eajournals.org; http://www.eajournals.org/wp-content/uploads/Statistical-Analysis-Internal-Consistency-Reliability-and-Construct-Validity.pdf.

[36] https://www.surveysystem.com/sscalc.htm. www.surveysystem.com, https://www.checkmarket.com/sample-size-calculator/ [Internet]. 2019. Available from: https://www.surveysystem.com/index.htm. Accessed Feb 2018.

[37] Health statistical department, planning and computer department U ministry of health. Annual Statistical Book 2017 G 1438. Cochrane Database Syst Rev. 2017;(2):142. https://www.moh.gov.sa/en/Ministry/Statistics/book/Documents/book-Statistics.pdf. Accessed 28 June 2021.

[38] Tong A, Synnot A, Crowe S, Hill S, Matus A, Scholes-Robertson N (2019). Reporting guideline for priority setting of health research (REPRISE). BMC Med Res Methodol.

[39] Sector NC for N-P. Civil Society Partnership [Internet]. Available from: https://ncnp.gov.sa/en [Accessed June 2022].https://www.my.gov.sa/wps/portal/snp/eParticipation/civilSocietyPartnership/!ut/p/z0/04_Sj9CPykssy0xPLMnMz0vMAfIjo8zi_QxdDTwMTQz9LXyNTA0CzYx8PR39vDw9_cz1g1Pz9AuyHRUBbX9cww

[40] Government of Ireland. Research Priority Areas 2018 to 2023. Innov 2020 [Internet]. 2018;26. Available from: https://dbei.gov.ie/en/Publications/Publication-files/Research-Priority-Areas-2018-to-2023.pdf [20 Mar 2019].

[41] Remme JHF, Adam T, Becerra-Posada F, D’Arcangues C, Devlin M, Gardner C, et al. Defining research to improve health systems. PLoS Med. 2010;7:1–6. 10.1371/journal.pmed.1001000 PMC2993153 21124816

[42] Don de Savigny, Harun Kasale, Conrad Mbuya and GR. Fixing health systems, 2nd edn. Ottawa, Canada: The International Development Research Centre; 2008. https://www.idrc.ca/en/book/fixing-health-systems-2nd-edition. Accessed 21 June 2021.

[43] Commonwealth Secretariat. A Systems Framework for Healthy Policy Advancing, Global Health Security and Sustainable Well-being for All. the Commonwealth Secretariat. Marlborough House. 2016. Available from: https://www.thecommonwealth-healthhub.net/sfhp/. Accessed Feb 2019.

[44] WHO. Monitoring the building blocks of health systems: a handbook of indicators and their measurement strategies. 2010. p. 8–11.

[45] World Health Organization. Improving the quality of health services—tools and resources. WHO Service Delivery and Safety Department. 2018. 1–59 p.

[46] Rockville M. Health Systems Research. [Internet]. Health Systems Research. Agency for Healthcare Research and Quality. 2020. Available from: https://www.ahrq.gov/healthsystemsresearch/index.html [Accessed Feb 2019].

[47] World Health Organization (2017). A strategic framework for health workforce development in the Eastern Mediterranean Region. East Mediterr Health J.

[48] Monday Hris B on. What is a Health Information System? [Internet]. Data inside Digital Guardian’s Blog. 2020. Available from: https://digitalguardian.com/blog/what-health-information-system [Accessed Feb 2019].

[49] Rouse MSW and AD. Health Information Technology Office for Civil Rights Headquarters U.S. Department of Health and Human Services [Internet]. TechTarget 20. 2019. Available from: https://searchhealthit.techtarget.com/definition/Health-IT-information-technology [Accessed Feb 2019].

[50] European Commission. eHealth action plan 2012–2020: innovative healthcare for the 21st century. Communication from the commission to the European parliament, the council, the European economic and social committee and the committee of the regions. Brussels, 6.12. 2012. 2012.Available from: https://ec.europa.eu/digital-single-market/en/news/ehealth-action-plan-2012-2020-innovative-healthcare-21st-century [Accessed Feb 2019].

[51] Barbabella F, Melchiorre MG, Papa R, Lamura G. How can eHealth improve care for people with multimorbidity in Europe? Health Systems and Policy Analysis. 2016; p. 9–15. (ISSN: 1997-8073). https://pubmed.ncbi.nlm.nih.gov/29144695/; https://www.ncbi.nlm.nih.gov/books/NBK464571/; https://www.euro.who.int/__data/assets/pdf_file/0007/337588/PB_25.pdf. Accessed 18 June 2021. 29144695

[52] Kazi DS (2016). From innovation to implementation. J Am Coll Cardiol.

[53] World Health Assembly, 72. Access to medicines and vaccines: report by the Director-General. World Health Organization. 2019. https://apps.who.int/iris/handle/10665/328625. Accessed 20 June 2021.

[54] WHO. Health financing [Internet]. WHO. 2020. Available from: https://www.who.int/health-topics/health-financing#tab=tab_1[Accessed Oct 2019].

[55] WHO. Cost effectiveness and strategic planning (WHO-CHOICE) [Internet]. 2020. Available from: https://www.who.int/choice/en/ [Accessed Oct 2019].

[56] WHO. Health system governance [Internet]. WHO. 2020. Available from: https://www.who.int/health-topics/health-systems-governance#tab=tab_1[Accessed Oct 2019].

[57] WHO. Health Emergency and Disaster Risk Management: Overview [Internet]. Health Emergency and Disaster Risk Management Fact Sheets. 2019. 48 p. Available from: https://www.who.int/hac/techguidance/preparedness/health-emergency-and-disaster-risk-management-framework-eng.pdf?ua=1[Accessed Oct 2019].

[58] Memish ZA, Jaber S, Mokdad AH, AlMazroa MA, Murray CJL, Al Rabeeah AA, et al. Burden of disease, injuries, and risk factors in the Kingdom of Saudi Arabia, 1990–2010. Prev Chronic Dis [Internet]. 2014;11:E169. 10.5888/pcd11.140176 PMC4184091 25275806

[59] Khalil MKM. Integrative medicine: the imperative for health justice in the other side of the world. J Alternative Complementary Med 2018;24:101–103. 10.1089/acm.2018.0128 29792515

[60] Harvard T.H. Chan School of Public Health 2020.The Nutrition Source. Disease Prevention [Internet]. Harvard T.H. Chan School of Public Health 2020. 2020. Available from: https://www.hsph.harvard.edu/nutritionsource/disease-prevention/ [Accessed June 2020].

[61] Promotion OODPAH. Oral Health, U.S. Department of Health and Human Services 2020 [Internet]. The U.S. Department of Health and Human Services. 2020. Available from: https://www.healthypeople.gov/2020/topics-objectives/topic/oral-health [Accessed May 2019].

[62] World Health Organization. Pharmaceutical products. Available from:http://www.emro.who.int/health-topics/pharmaceutical-products/index.html [Accessed Jan 2020].

[63] Golfomitsou S, Katrakazis T, Heritage A. The role of educators in promoting collaborative research. CeROArt. 2017;(HS).

[64] Research TC of Sand. Health research priorities in Sultanate of Oman [Internet]. Ministry of Health in Sultanate of Oman. 2014. Available from: https://mohcsr.gov.om/wp-content/uploads/2015/03/research-priority-8.4.2014.pdf [20 Mar 2019].

