# Supplementary material for: Health Research Priority Agenda for Ministry of Health, Kingdom of Saudi Arabia from 2020 to 2025
Source: J Epidemiol Glob Health. 2022 Oct 4;12(4):413–29. doi: 10.1007/s44197-022-00061-5 (PMC9531642; doi:10.1007/s44197-022-00061-5)
Supplement: Supplementary file 1 — Supplementary file1 (PDF 365 KB) [file 44197_2022_61_MOESM1_ESM.pdf]

Title of Study: Setting Research Priority Agenda for MoH, KSA, 2019-2023G

Researchers : Dr. Athari Alotaibi, Dr. Maha Alosaimi and Dr. Wafaa Saleh

The General Directorate of Research and Studies **(GDRS)** has the pleasure to invite you to participate in the current research study by completing the relevant questionnaire about prioritizing health research at the Ministry of Health (MoH). The study objectives are to; identify priorities that will maximize research impact on population health, reduce duplication of effort, and promote collaboration.

1. I confirm that I have read and understood the information sheet for the current study. I had the opportunity to look at the information, ask questions, and receive satisfactory answers as appropriate.
2. I understand that my participation is voluntary and I am free to withdraw at any time without giving any reason or claim any legal rights.
3. I understand that the research team will review the relevant parts of the data collected during the study, as it is beneficial to my community and my country so, I would like to participate in this research. I give permission to these individuals to obtain my information through my trust in them.

It is estimated to complete the questionnaire in about 10 minutes.

☐ I have read and understood the points and statements of this form. I have had all my questions answered to my satisfaction, and I voluntarily agree to participate in this study.

Start

Section A — Demographic Information | Section B — Official Information | Section C — Options for priority research topics

Email address: *

Write your email address

Age: *

Gender: *

Nationality: *

Educational level: *

The region / province in which you are currently working: *

Section A
Demographic Information

Section B
Official Information

Section C
Options for priority research topics

Please write the name of the institution / administration / association which you are affiliated to: *

Job / Position: *

Professional Classification: *

Professional Specialty: *

Health specialties: *

Current Job/ position title: *

Duration in the current job / position: *

0

Please clarify your influence in health policymaking process

◉ DIRECT ○ INDIRECT ○ Not applicable

Have you ever contributed to scientific research

○ Yes ◉ No

If yes, please mention the number of research projects:

0

Have you ever been an author or coauthor of published research papers

○ Yes ◉ No

If yes, please indicate the number of published papers:

0

C.I. Write the most important research topics in health fields that you want to be included in MoH research priority agenda

(The answer to this question is mandatory)

C.I.1. Choose general field / topic

Write Specific topics / titles

1.1.1 -

1.1.2 -

1.1.3 -

Please for each selected general research topic, choose the appropriate rating of the criteria listed below based on the included rating metrics

Availability of pre-existing data

Ethical acceptability

The burden of the research topic

Equity focus and community concern /demand

Cost justification

Capacity of the system to undertake the research

The research will lead to overall reduction of the burden

Chances of implementation of the research outcome

Urgency for the data needed for decision making

Opportunity to strengthen collaboration with partners

C. I.2. Would you like to add another general research priority topic?

○ Yes ◉ No

C.II. Write the most important research topic as regards Ministry of Health's strategic objectives of the National Transformation 2020 program and vision of the Kingdom 2030 ,that you prefer to be included in MoH research priority agenda
(The answer to this question is optional)

C.II.1. Choose general field / topic

Write Specific topics / titles

2.1.1 -

2.1.2 -

2.1.3 -

Please for each selected general research topic, choose the appropriate rating of the criteria listed below based on the included rating metrics

Availability of pre-existing data

Ethical acceptability

The burden of the research topic

Equity focus and community concern /demand

Cost justification

Capacity of the system to undertake the research

The research will lead to overall reduction of the burden

Chances of implementation of the research outcome

Urgency for the data needed for decision making

Opportunity to strengthen collaboration with partners

C.II.2. Would you like to add another general research priority topic?

○ Yes ◉ No

C.II.1. Choose general field / topic, include:

- Public Health.
- Road Traffic Accident (RTA).
- Value-Based Health care.
- Health care access.

C.III. Write the appropriate research topics that you prefer to be included as national and international collaborative research topics
(The answer to this question is optional)

Write Specific topics / titles

3.1.1 -

3.1.2 -

3.1.3 -

Please for each selected general research topic, choose the appropriate rating of the criteria listed below based on the included rating metrics

Availability of pre-existing data

Ethical acceptability

The burden of the research topic

Equity focus and community concern /demand

Cost justification

Capacity of the system to undertake the research

The research will lead to overall reduction of the burden

Chances of implementation of the research outcome

Urgency for the data needed for decision making

Opportunity to strengthen collaboration with partners
