# Supplementary material for: Health Research Priority Agenda for Ministry of Health, Kingdom of Saudi Arabia from 2020 to 2025
Source: J Epidemiol Glob Health. 2022 Oct 4;12(4):413–29. doi: 10.1007/s44197-022-00061-5 (PMC9531642; doi:10.1007/s44197-022-00061-5)
Supplement: Supplementary file 2 — Supplementary file2 (DOCX 93 KB) [file 44197_2022_61_MOESM2_ESM.docx]

# Agenda 1: Health System Research Priority Themes

Health System Research Domain addresses health system and policy questions that are not disease-specific but concern systems problems that have repercussions on the performance of the health system as a whole. It addresses a wide range of questions, from health financing, governance, and policy to problems with structuring, planning, management, human resources, service delivery, referral, and quality of care in the public and private sector. This research can provide a robust and accurate understanding of health systems challenges and their potential solutions, thereby improving the utility of the findings in other settings.(1)

Health systems research by necessity is highly multidisciplinary, with a strong emphasis on social sciences, economics, and anthropological investigations. Much ongoing research consists of descriptive, comparative, and evaluation studies and secondary analytical research. Although experimental studies are less common, partly because of operational and ethical challenges in experimenting at the health system level, they can be very informative and provide convincing evidence on the benefit of innovations in health system efficiency and health impact.(2)

The proposed goals of Kingdom of Saudi Arabia, Health Sector Transformation Strategy are; firstly, to improve health: increase the length, wellbeing and quality of life of Saudi citizens, which includes the Vision 2030 goal of increasing the life expectancy of citizens to 80 years by 2030. Secondly, to improve healthcare: by improving the quality and consistency of services and the performance and accountability of healthcare organizations and staff to deliver care that is safe, effective, patient-centered, timely and equitable. Thirdly, to improve value: by containing costs, improving outcomes, controlling public healthcare expenditure and guiding new investment. All three transformation goals conform with, and are enablers of, the Vision 2030 strategic objectives for health: access, value and public health.(3)

Health systems are already struggling to meet current demands and the challenges facing society over the next 40 years will add further stress. As a result, solutions may require more innovative approaches that address the wider determinants of health and require multi-disciplinary partnerships, which are traditionally outside the remit of health policy-makers. The environmental determinants, which include climate change, disasters and emergencies, interact with and potentially have an impact on the social and economic determinants of health, including an ageing population and economic decline in many parts of the world. (4)

WHO framework describes health systems in terms of six core components or “building blocks”: (i) service delivery, (ii) health workforce, (iii) health information systems, (iv) access to essential medicines, (v) financing, and (vi) leadership/ governance. The six building blocks contribute to the strengthening of health systems in different ways. Some cross-cutting components, such as leadership/governance and health information systems, provide the basis for the overall policy and regulation of all the other health system blocks. Key input components to the health system include specifically, financing and the health workforce. A third group, namely medical products and technologies and service delivery, reflects the immediate outputs of the health system, i.e. the availability and distribution of care. (46) **(figure 5)**

WHO addresses improving the quality of health services and highlights research as one of the adaptable tools and resources that can support local quality improvement efforts. It aims to support implementation of quality improvement approaches to make health services more effective, safe and people centered. There is an increasing collective recognition that quality health services should be; effective, safe, people-centered, timely, equitable, integrated and efficient. (5) Health systems research provides evidence that, when applied can make healthcare affordable, safe, effective, equitable, accessible, and patient-centered. AHRQ invests in research to generate new evidence to help health care systems and health care professionals improve the lives of the patients they serve. (6)

Good service delivery is a vital element of any health system. Service delivery is a fundamental input to population health status, along with other factors, including social determinants of health. The network of service delivery in any well-functioning health system should have the following key characteristics; comprehensiveness, accessibility, coverage, Person-centredness continuity, quality, coordination, accountability and efficiency.(7)

The Framework for action for health workforce development in the Eastern Mediterranean Region has been developed in response to the health workforce challenges facing the Region, in line with Sustainable Development Goal target 3.C to increase the recruitment, development, training and retention of the health workforce. It is imperative that the Region strives towards institutionalizing strategic planning as an approach in responding to health workforce challenges. The framework aims to guide country and regional action to strengthen the health workforce to ensure access for all people to an adequate, competent, well balanced, motivated and responsive health workforce which contributes to health systems strengthening and progress towards universal health coverage in the Region.(8)

A health information system (HIS) refers to a system designed to manage healthcare data. This includes systems that collect, store, manage and transmit a patient’s electronic medical record (EMR), a hospital’s operational management or a system supporting healthcare policy decisions. Health information systems also include those systems that handle data related to the activities of providers and health organizations. Data is analysed to improve patient outcomes, inform research, and influence policy-making and decision-making.(9)

Health information technology (health IT) involves the exchange of health information in an electronic environment.  Widespread use of health IT within the health care industry will improve the quality of health care, prevent medical errors, reduce health care costs, increase administrative efficiencies, decrease paperwork, and expand access to affordable health care.  It is imperative that the privacy and security of electronic health information be ensured as this information is maintained and transmitted electronically.(10)

eHealth has been defined by the European Commission as “the use of Information and Communication Technologies (ICT) in health products, services and processes combined with organizational change in health care systems and new skills, in order to improve health of citizens, efficiency and productivity in health care delivery, and the economic and social value of health”.(11) eHealth is an umbrella term that covers a wide range of health and care services delivered through information and communication technologies (ICTs), such as electronic health records (EHRs), health information systems, remote monitoring and consultation services (e.g. telehealth, telemedicine, telecare), tools for self-management, and health data analytics. Health is a subset of eHealth that is linked to mobile telephony and applications.(12)

eHealth has experienced a period of significant growth and maturity in recent years. Such investments are most often seen in the context of achieving health system reform, providing new and innovative modes of health care delivery or offering efficient methods of access and exchange of health information. Most notable, however, is the transition of eHealth to a subject of strategic importance for policy-makers.(13)

Improving access to health products is a multidimensional challenge that requires comprehensive national policies and strategies. These should align public health needs with economic and social development objectives and promote collaboration with other sectors, partners and stakeholders; they also need to be aligned with legal and regulatory frameworks and cover the entire product life cycle, from research and development to quality assurance, supply chain management and use.(14)

Development of innovation ecosystem is a substantial approach to ensure that enterprises can collaborate with researchers and to engage effectively in areas of impact to innovate and develop new products, services, solutions and new business models to underpin leadership positions in international markets and to meet global and societal challenges. (14) In an era of ageing populations and growing health expenditures, medical innovations are needed to improve the quality of people’s lives and increase the efficiency of health care systems and contribute to significant cost savings and economic growth.(15)

WHO 2019 road map supports countries’ ability to allocate resources more effectively through evidence-based decisions to ensure that cost-effective health products are included in a country’s essential medicines list, essential diagnostics lists or reimbursement lists and through more efficient procurement and supply processes and rational use of medicines. Support for fair pricing and policy implementation to reduce out-of-pocket expenditures. There is a pressing need to improve access to timely, robust and relevant information concerning health products. Unbiased information that is free of any conflict of interest is vital for the sound selection, incorporation, prescription and use of health products. (14)

Health financing is a core function of health systems that can enable progress towards universal health coverage by improving effective service coverage and financial protection. Today, millions of people do not access services due to the cost. Many others receive poor quality of services even when they pay out-of-pocket. Carefully designed and implemented health financing policies can help to address these issues. For example, contracting and payment arrangements can incentivize care coordination and improved quality of care; sufficient and timely disbursement of funds to providers can help to ensure adequate staffing and medicines to treat patients.(16) Cost-effectiveness, costs and strategic planning can help guide policy decisions to ensure that money spent on health is allocated in a way that the greatest possible health outcomes are achieved in the most feasible manner.(17)

Leadership and governance involves ensuring strategic policy frameworks exist and are combined with effective oversight, coalition-building, regulation, attention to system-design and accountability. Three main categories of stakeholders who interact with each other determine the health system and its governance:

- the State (government organizations and agencies at central and sub-national level);
- the health service providers (different public and private for and not for profit clinical, para-medical and non-clinical health services providers; unions and other professional associations; networks of care or of services);
- the citizen (population representatives, patients’ associations, CSOs/NGOs, citizens associations protecting the poor, etc.) who become service users when they interact with health service providers.(18)

All communities are at risk of emergencies and disasters including those associated with infectious disease outbreaks, conflicts, and natural, technological and other hazards. The health, economic, political and societal consequences of these events can be devastating. Climate change, unplanned urbanization, population growth and displacement, antimicrobial resistance and state fragility are contributing to the increasing frequency, severity and impacts of many types of hazardous events that may lead to emergencies and disasters without effective risk management. Health emergency and disaster risk management (EDRM) emphasizes assessing, communicating and reducing risks across the continuum of prevention, preparedness, readiness, response and recovery, and building the resilience of communities.(19)

WHO's Emergency, Trauma and Acute Care program is dedicated to strengthening the emergency care systems that serve as the first point of contact with the health system for so much of the world, and to supporting the development of quality, timely emergency care accessible to all.(19)

World Health Assembly committed to recognize Emergency care a key health; as better and faster services for time-sensitive health conditions, including injuries, heart attacks, mental health conditions, infections or pregnancy complications.

A functional emergency care system is essential to universal health coverage, and investing in frontline care saves lives, increases impact and reduces costs in other parts of the health system. As timeliness is an essential component of quality care and that millions of deaths and long-term disabilities could be prevented if emergency care services exist and patients reach them in time. Steps agreed to strengthen countries’ emergency care include developing policies for sustainable funding, governance and universal access to emergency care for all, and integrating emergency care into health delivery and training strategies at all levels. The application of WHO emergency care system assessment helps to identify gaps and context-relevant priorities.(20)

Planning for Coronavirus Disease 2019 (COVID-19) pandemic is critical for maintaining healthcare services during a response. Sufficient health system and public health capacities should be in place to enable the major shift from detecting and treating cases to detecting and isolating all cases; Detection: suspect cases should be detected quickly after symptom onset through active case finding, self-reporting and entry screening; Testing: all suspected cases should have test results within 24 hours of identification and sampling, and to verify the virus-free status of patients who have recovered; Isolation: all confirmed cases should be effectively isolated immediately and until they are no longer infectious; Quarantine: all close contacts should be traced, quarantined and monitored for 14 days.(21)

1. **Health Service Delivery**
2. **Networks of Comprehensive , Integrated and Equitable Care**

- **Coordination, Coverage and Continuity**
- PHCCs : How to prepare PHCCs to diagnose and manage internal medicine diseases as, recurrent UTI
- Development of performance and broadening specialties of primary health care centers services
- New models of PHC and multi-specialty clinics
- Emergency (ER) cases vs PHC cases and reasons for going to ER rather than PHC.
- Reasons for lack of effective health centers in service of community and the conversion of all cases to hospitals
- Communication between the primary health centers and the hospitals
- Pregnancy care in primary health care centers
- Maternal-fetal medicine (MFM) update and future of maternity care
- Ways of developing therapeutic and surgical care for children
- Pressure ulcer and pain management nursing care plan
- Family medicine / Expand services within clinics
- Patients with extended care and Long-stay patients in hospitals
- Impact of patient education on hospital readmission frequency
- Outcomes of critically ill patients and reduction of long stay in critical unit
- Health care records – Documentation and Management; Extensive paperwork documentation without reassessment of the form necessity
- Health services management and bed management system
- Model of care and case report
- Implementing collaborative Team Based Care will help improve Sequel’s outcomes in Care Coordination
- Effectiveness of online application users; number of Tahour application at KFMC
- Implementation of health education and preventive care programs
- Employee education about the study before making a decision for any health services
- Operational efficiency, patient composition and spatial context of health centers in the Kingdom of Saudi Arabia
- Improve and develop the internal environment of the health facility at KSA
- Disparities in health
- **Ambulance Services and Transportation**
- Air and ground ambulance services and transportation
- Inter-facility integration between ambulance services and hospitals
- **Out of Saudi Arabia Care**
- Medical referrals to out of the kingdom and out of region
- Organ Transplant out of the Kingdom
- **Family Medicine & Home Care Services**
- Accessibility of extended care such as rehabilitation long term home care and to improve quality of life and Electronic solutions in home health care access.
- Barriers of working at home care by nurses and faced challenges
- Home Total Parenteral nutrition (TPN)

- **Health Gathering Haj**

1. **Quality of Care**

- **Safety**
- Cost effectiveness of the current infection control methods applied and the economic burden
- Increase health care providers’ awareness and practice about infection control
- Safe methods for disposal of medical wastes
- Patient safety in the neonatal intensive care unit (NICU)
- Safety in Labor and delivery room
- Measure the knowledge of safety among the employees at MOH
- Comparison of knowledge, attitude, and practices regarding needle-stick injury (NSI) among health care providers and management
- Infection control; improving patient and health practitioners’ safety
- Bacterial and viral nosocomial infection
- Bacterial Contamination of Electronic Tablet Devices Used by Resident Physicians
- Occupational health for citizens & immigrants’ staff and facility visitors’ health
- Work place environment of the health care facility at KSA
- The effect of Business Process Enterprise Architecture on advancing the work place
- Probability of risk in health facilities and the potential of unexpected fire
- **Effectiveness**
- Quality of care indicators, Quality metrics and risk
- Confidence of quality care in Primary Healthcare Centers, and Diabetes Management Centers & the proper availability of qualified staff, services and materials
- Pharmacotherapy quality improvement; pharmacist counseling in pharmacotherapy quality improvement:
- Enhancement patients care and medications compliance
- Reduction of prolonged hospital stay
- Reduction in Hospitals’ Readmission Rates
- Among hemodialysis patients
- The accuracy of the readings of the devices - Cost and quality
- Study of higher potentials that serve the needs of the physicians to monitor and follow up inpatients
- Knowledge, attitude, and practice toward evidence-based medicine (EBM) among health care providers
- **People-Centeredness; Patient Satisfaction**
- Study of reasons of unsatisfied patients discharged from ministry of health hospitals and seeking additional medical care in private medical centers
- Patient feedback about delivery room
- Effective communication of patients with service providers
- Ways to Provide wide range services and customers' satisfaction with services
- The effect of patient education on patient satisfaction
- Assessment of patient satisfaction with the provided services in emergency departments
- Patient experience in health services
- Patient ownership at the level of Primary Healthcare and Diabetes Centers
- **Value-Based Healthcare**
- Efficiency and effectiveness of value based healthcare in the health system
- limited Value-based healthcare research
- How to formulate the Value Based care concept in our healthcare system
- Implementation of the value based healthcare concept, models and cost management
- Defining value from the population and social perspective and measuring health and healthcare outcomes
- Value based concept with regard expensive medication in MOH
- **Timely**
- Effective ways to reduce patient waiting times
- Effect of prescription waiting time on patient satisfaction mediated by service quality of hospitals’ pharmacy unit
- Patients’ response to waiting time in an outpatient department (OPD) pharmacy
- **Key Performance Indicators**

- Data and performance analysis
- Effect of organizational structure on performance
- key performance indicators (KPIs) as an integral tool for measuring quality

1. **Accountability and Medical Errors**

- Compliance to, and effectiveness of the implementation of clinical practice guidelines
- Medical errors related to poor endorsement among mergency medicine staff
- Child maltreatment
- Accountable Care Organization

1. **Health Service Research**

- Bridging the gap between research and practice
- Clinical trials and new drugs innovation
- Research in antimicrobial resistance
- Customer relationship management research in hospitality industry: a review and classification
- Research awareness of taff in KSA, based on guidance and motivation Program towards achieving health research culture.
- Evaluation of current status of oral diseases prevention programs
- Randomized controlled trial ; Recovery from critical illness by improving basic, therapeutic nutrition & protein intake
- Measuring the impact of health campaigns and MOH campaigns among the community during 2018
- Plain packing with Austrian campaign and illicit trade in tobacco products among the gulf region

1. **Health Workforce**
2. **Health Workforce Production**

- Staff Qualification. Qualifications and skills of hospital & PHC Directors
- MoH approval of all qualifications issued by Saudi Universities

1. **Health Workforce Governance**

- Measuring training return for skill development of hospital managers and staff
- Capability building - Economic impact - Strategic impact
- Workforce planning and organizational Commitment
- Pharmacist clinical specialists development; The impact of and challenges facing application of programs to share best practices and optimize patient care within the unique area of specialty pharmacy
- Assess, develop and fulfill human resources’ program and services

1. **Availability, Accessibility and Quality**

- Physicians & health workforce to population ratios by numbers and specialties, and distribution of health facilities across all regions to ensure equitable health care
- Availability of specific health care specialties
- Medical talent acquisition & retention and staff turnover
- The impact of staff development techniques on creativity
- The distribution of health specialties across health facilities
- Prevalence of burnout syndrome among health-care professionals and in ED
- The effect of sick leave and low workflow
- Administrative factors affecting Saudi employees' absence
- The Impact of heavy perceived hospital staff workloads and different work environment pressures on quality and safety of care provided and patient outcomes
- Advancing nursing career & specialization and impact of long working hours and shift work
- Delegation of nurses in healthcare according to the job description
- Blood Bank Nurses
- Improve human resources to provide high quality services

1. **Health Workforce Motivations**

- Depression among medical residents’ board
- Manpower Support and how to increase satisfaction of staff in ED
- Impact of job satisfaction and incentives on staff performance and quality of care

1. **Health Workforce Development ; Medical Education and Training**

- Centers of Excellence (COE) for Health Professionals Training (CHPT)
- Improving Standards in Health Professional Education
- Funding for health training
- Leadership in medical education
- Accredited training of the trainers (TOT) courses
- Accreditation of Medical Education Institutes and programs
- An approach to integrating interprofessional education and implementing collaborative Team Based Care will help improve Sequel’s outcomes in Care Coordination
- Teaching methods

1. **Health Information and Communication Technologies’ Systems**

- Health transformation and automation in healthcare system
- Health technology system in Saudi Arabia
- Use open source to improve the performance of information recovery systems
- Health Statistics in collaboration with General Authority for Statistics and Bio indicators
- Artificial intelligence applications for information retrieval and concerns about it
- Central platform for health records and information
- Translational bioinformatics and basics of medical statistics and health indicators
- The importance of and accessibility to information and data analysis
- Knowledge and practices concerning new technology of health care referral system
- Accreditation of all medical procedures provided to the patient electronically
- Electronic versus manual medical files and data processing
- Establishing database for outcomes of care in birth centers
- Importance of establishing a national blood donor database in improving blood bank services; reducing time, money and risk
- Accessibility to information to reduce time & effort and implement wise decisions
- Access to one file for all patients in both public and private health centers
- Activating electronic transactions
- Computational pathology and case report about rare diseases
- Health care records and documentation management
- Promote and share health information as support system for clinical decisions
- E- Health information exchange policy; patient privacy, security, safety and ethics

1. **Access to Healthcare, Essential Medical Products, Vaccines and Innovative Approaches and Technologies**
2. **Access to Healthcare**

- Access to primary health care centers and increasing numbers of visits to the PHC
- Preventive Care and access to enough number of health centers
- Accessibility to extended care such as rehab. long-term care and home health care to improve the individual’s quality of life and population health
- Easy access to mental health services & available appointment for follow up
- Easy access to early health care appointment and reasons of late hospitals’ appointment
- Emergency patients’ time access to care and specialized healthcare access
- Barriers to access health services in the main and peripheral areas
- The effectiveness of mobile clinics

1. **Access to Essential Medical Products**

- Improving the quality and quantity of medical stocks
- Access to pharmaceutical care in MOH
- Impact of medication among Saudi Population

1. **Access to Vaccines**

- Vaccination programs and general pediatric care

1. **Innovative Approaches and Technologies**

- Bone marrow transplantation (BMT), and CAR T-cell therapy as a new approach to cancer treatment
- Devices and Drug development
- Development of viral vaccines

1. **Health Systems Financing and Economics**

- Health systems financing and economics
- Financial management in healthcare organization
- Efficiency of spending in public hospitals and improving business processes
- Economic impact in health system
- Population health and economic cost accountable of model
- Evaluation of economic impact and analysis of complementary medicine approaches as a step for integration into main healthcare system
- Cost effectiveness analysis of the national screening program for breast cancer in KSA
- Cost of knee injuries in Health care system
- Cost effective Strategies for the prevention of blindness and vision impairment in Saudi Arabia
- Patient management by turn on laboratory services’ provider into an investment Partner for more success
- Purchasing services of radiology, laboratory, laundry and sterilization

1. **Health System Governance and Leadership**

- Transforming and modernization of the Governance
- Health system policies, management & monitoring
- Evaluating the health and economic impact of health policies & initiatives on society
- Impact evaluation of the health risks prevention programs, carried out by MoH during the past ten years and determining knowledge & awareness impact and community response to these programs
- Collaboration between ministry of health and British agency of Care Quality Commission to come out with regulations for plastic surgery
- Improve and standardize operational policies and procedures in health sectors
- Partnership tools between government and other civil parties and roll of nonprofit organizations in providing health services
- Sustainable Development Goals (SDGs)
- WHO 90 90 90 goal for HIV diagnosis, treatment and viral suppression
- Medical planning, equity of health care and evidence based medical design
- Enhancement of Autism Centers in Saudi Arabia
- Providing beds, appointments and the treatment for the patients and improving health prevention
- Leadership in healthcare, change management and problem solving
- Concept of leadership style and challenges facing women leaders
- Impact of performance evaluation on timely decision-making and health system improvement
- Healing environment and internal design among the health facilities
- Adoption of interventions to address non-communicable diseases; cancer overall
- Spiritual support program in Stamford in the USA
- Track of Counterfeit medication
- Public health in all policy
- Road traffic law enforcement
- Introduce public health law and establish public health laboratory
- Development of Primary Eye Care in Saudi Arabia
- Development of school eye screening and the National Program for Control of Blindness
- Policy to increase tax for junk food and lower the healthy food prices in KSA
- Shared health
- Application of health insurance for all individuals in KSA similar to the UAE
- Volunteer’s management in health field

1. **Health System Preparedness and Response to Disasters and Emergency Care**
2. **Emergency Care**

- **First Aid**
- **Triage**
- Triage assessment by designated experts to properly screen critical cases
- The effectiveness of Canadian Triage and Acuity Scale in minimizing cold cases in ER
- **Emergency (ER) Department Services**
- Development to serve patients & medical staff and assessment of patient care outcome
- Educate and support emergency medical staff to manage patients in an efficient way
- Glasgow Outcome Scale (GOS) and GOS scores
- Turnaround times of laboratory tests for (ER) patients, & waiting times as well
- Time factor in management of critical cases
- Palliative care and end-of-life care
- policy of urgent care in neurosurgery
- Patient information of neurosurgical degenerative spine
- ER criteria for patients visits & study of length of stay of the patient in intensive care unit and in wards
- Insufficient capacity for critical care of children
- Benefits of Integration between PHC and ER departments
- How to increase the knowledge of the general doctors in primary healthcare in hot topics in medicine
- Benefits of continuing medical education conferences & increasing training projects for better performance of emergency services provider staff
- Decrease time of waiting in Emergency

1. **Disasters, Epidemics and Pandemics**

- Phases of the disaster management cycle: prevention, mitigation, preparedness, response, rehabilitation, reconstruction and recovery.
- Environmental, nuclear, radiation, biological and chemical disasters
- Emerging infectious diseases & Preparedness and response for public health emergency
- Response of health care providers and governorate to medical disaster
- Scientific and practical training of all medical provider staff.

# Agenda 2: Diseases, Health Problems, Public Health and Medical Care Themes

Health Sector Transformation Strategy of Kingdom of Saudi Arabia clarified that rates of avoidable injury and non-communicable disease remain high by regional and international standards. The Kingdom has made notable progress in improving the health of its population over recent decades, particularly in areas of child and maternal mortality and the reduction of communicable disease. For both males and females, life expectancy at birth improved from 64 years in 1970 to 75 years in 2015. There are targets set to ensure it increases to 80 years by 2030. There is considerable scope to reduce avoidable mortality and morbidity in both the working and elderly populations. Particular areas of concern include heart disease, stroke, diabetes mellitus, respiratory disease, mental health, road traffic accidents and congenital diseases, all of which are amenable to reduction. There is a need to strengthen the prevention of non-communicable disease and injury thereby reducing avoidable illness and death. The risk of major outbreaks of communicable disease also remains substantial, especially at Hajj or following natural or man-made disasters.(22) KSA is facing a rising burden of non-communicable diseases and road traffic injuries as a result of rapid changes in behaviors. There is a massive need for major intervention to reduce these burdens and to engage other sectors of the government and the community in these efforts.(23)

Reforming the mode of healthcare financing should not be the only goal of healthcare transformation in Saudi Arabia. Transformation should also include a move toward integrative health and medicine and to promote the culture of wellness.(24)
Saudis are more likely to seek healthcare only when they are sick, which may be too late in the face of the lifestyle diseases epidemics.(25)  More investment is needed in behavior change and to promote self-responsibility because well-being is not only performed by a social and professional health practice but is also informed by our own self-care and resilience.(26)  Investment in behavior change and well-being outside the boundaries of the healthcare system in the Saudi 2030 vision will have more impact on health and wellness of the Saudi citizen than direct spending on healthcare facilities.(27)

Chronic diseases —including [heart disease](https://www.hsph.harvard.edu/nutritionsource/disease-prevention/cardiovascular-disease/), [stroke](https://www.hsph.harvard.edu/nutritionsource/disease-prevention/cardiovascular-disease/cvd-types/), [diabetes](https://www.hsph.harvard.edu/nutritionsource/diabetes-full-story/), and cancer— account for some of the most common health problems in the United States, according to statistics from the Centers for Disease Control and Prevention (CDC). Yet many of these chronic diseases are preventable, as they’re linked to poor diet and lifestyle choices including tobacco use, excessive alcohol consumption, and inadequate physical activity.(28)

Malnutrition has been researched and addressed within two distinct silos, focusing either on undernutrition, food insecurity, and micronutrient deficiencies, or on overweight, obesity, and dietary excess. Long-lasting effects of malnutrition in early life can be attributed to interconnected biological pathways, involving imbalance of the gut microbiome, inflammation and metabolic dysregulation. Life-course exposure to early undernutrition followed by later overweight increases the risk of non-communicable disease, and in women increases the risk of childbirth complications. These life-course trajectories are shaped both by societal driving factors—ie, rapidly changing diets, norms of eating, and physical activity patterns—and by broader ecological factors such as pathogen burden and extrinsic mortality risk.(28) The World Health Organization (WHO) addressed the double burden and the global risks of malnutrition in all its forms, which threaten the economic, social and environmental health of individuals, families, entire communities, countries and the planet. WHO intends to accelerate political commitment and advance the achievement of the SDGs, especially SDG 2 and SDG 3.(29)

Humans interact with the environment constantly. These interactions affect quality of life, years of healthy life lived, and health disparities. WHO defines environment, as it relates to health, as “all the physical, chemical, and biological factors external to a person, and all the related behaviors.” Environmental health consists of preventing or controlling disease, injury, and disability related to the interactions between people and their environment. Globally, 23% of all deaths and 26% of deaths among children under age 5 are due to preventable environmental factors.(30)

The goal of oral health is to prevent and control oral and craniofacial diseases, conditions, and injuries, and improve access to preventive services and dental care. The health of the teeth, the mouth, and the surrounding craniofacial (skull and face) structures is central to a person’s overall health and well-being. Oral and craniofacial diseases and conditions include: dental caries (tooth decay), periodontal (gum) diseases, cleft lip and palate, oral and facial pain, oral and pharyngeal (mouth and throat) cancers, xerostomia (dry mouth). There are also social determinants that affect oral health. In general, people with lower levels of education and income, and people from specific racial/ethnic groups, have higher rates of disease. People with disabilities and other health conditions, like diabetes, are more likely to have poor oral health.(31)

New, innovative therapeutics make it possible to prevent, treat and possibly cure a wider range of diseases than before, delivering better clinical outcomes and improving quality of life for patients. Gene editing technologies like CRISPR-Ca9 could make it possible to stop disease development completely and help address genetic disease predispositions. Targeted drugs such as anti-obesity or dementia-preventing drugs, as well as immunotherapeutics to treat debilitating diseases, will increase the ability to address this growing healthcare challenge. Countering antibiotic resistance will be crucial to ensure effective drug treatment overall. Technologies such as anti-microbial resistance countering will be important to minimise and stop the spread of anti-microbial resistance.(15) Pharmaceutical products are a fundamental component of both modern and traditional medicine. It is essential that such products are safe, effective, and of good quality, and are prescribed and used rationally.(32)

Innovation in medical devices as a research priority area aims to contribute to the continued improvement of patient safety and outcomes at affordable cost. As developing medical device technologies will provide affordable transformative solutions for chronic diseases, such as heart disease, diabetes and musculoskeletal diseases. The Diagnostics research priority area should focus on the development of next-generation biomedical diagnostic devices for diagnosing disease and sustaining human health. Diagnostic products form a critical part of healthcare delivery, as enabling the early and accurate detection is vital in ensuring successful treatment, and reducing health costs. (15) Equitable access to health products is a global priority, and the availability, accessibility, acceptability, and affordability of health products of assured quality need to be addressed in order to achieve the Sustainable Development Goals, in particular target 3.8. Every disease management strategy requires access to health products for prevention, diagnosis, treatment, palliative care and rehabilitation.(14)

Electronic health records (EHRs) are real-time, patient-centred records that provide immediate and secure information to authorized users. EHRs typically contain a record of the patient’s medical history, diagnoses and treatment, medications, allergies and immunizations, as well as radiology images and laboratory results. They expand on the information in a traditional paper-based medical record by making it digital and thus easier to search, analyse and share with other authorized parties. An EHR system plays a vital role in universal health coverage by supporting the diagnosis and treatment of patients through provision of rapid, comprehensive and timely patient information at the point of care.(13)

All around the world, acutely ill and injured people seek care every day.  Frontline providers manage children and adults with medical, surgical and obstetric emergencies, including injuries and infections, heart attacks and strokes, asthma and acute complications of pregnancy.  Prioritising an integrated approach to early recognition and resuscitation reduces the impact of all of these conditions.(33) In the coming years, an increasing emphasis on primary care is expected to begin to improve the common problem of Emergency Departments (EDs) being treated as the gateway to the hospital. Moreover, promising trends in the development of simulation-based education, improvements in residency programs and curricula, and diversification of healthcare providers indicate that the rapid progress experience over the course of the last decade is ready to continue for the next decade and beyond. Roadmaps like the National Transformation Plan 2020 and the Saudi Vision 2030 have also begun to pave the way for future developments. It is now the task of Saudi EM physicians and healthcare policymakers to conduct practical research and implement data-drive, evidence-based policies, and procedures to guide efforts to move towards a more preventive and primary care healthcare model.(34)

1. **Non-Communicable Diseases (NCDs)**

- **Endocrine Diseases**
- Diabetes Mellitus (DM) & The extent and effectiveness of diabetic patients commitment to therapeutic nutrition guidelines and complications of incompliance
- Increased incidence, prevalence and disease burden of type 1& 2 DM in Saudi Arabia
- Management impact of Endocrine (DM) Erectile Dysfunction (ED) by primary health care clinics physicians
- Epidemiology and, effect of the new anti-diabetic medications in control of DM
- Modalities of DM prevention in Saudi Arabia
- Diabetic ketoacidosis (DKA) & DM Hypoglycemia
- Diabetic foot
- Endocrine disorders & syndromes
- Adrenal Insufficiency
- Prevalence of Hashimoto's Thyroiditis and Thyroid Disorders management
- Incidence of abnormal TSH level in hospitalized critically ill patients
- Missed diagnosis and treatment of Hyperparathyroidism
- Retinal examination in internal medicine for prevention of visual impairment
- **Cardiovascular Diseases**
- Acute Myocardial Infarction (MI), Cardiac Tamponade & Cardiovascular Diseases
- Prevalence of Acute Coronary Artery Syndrome in young adults
- Hypertension prevalence, complications, management, disease burden and prevention
- Comparison between Anti-Hypertensive medications and the best combination
- Neutrophil Function in Ischemic Heart Disease (IHD)
- Inducibility of Ventricular Arrhythmias at Electrophysiological Study (EPS), as predictive power for subsequent arrhythmic events.
- Interrelationship between liver and heart diseases
- Relation between morbid obesity and cardio- respiratory and other diseases
- Effects of fatty foods, soft drinks and energy drinks on patients with hypertension, stroke & heart diseases
- Kidney and diabetic diseases influence on the cardiovascular health
- Cardiovascular health in patients with the inflammatory joint disease; a genetic approach to understanding excess mortality
- Arterial stiffness in different population
- **Thromboembolism & Cerebrovascular Events**
- Venous Thromboembolism (VTE)
- Incidence of Deep Venous Thrombosis (DVT) in young females
- Prevention of DVT and death using routine ultrasound surveillance for DVT
- Evaluation of some new diagnostic modalities for DVT
- Treatment of DVT in patients with arrhythmia
- Pulmonary Thromboembolism ; epidemiology & management
- The association between Pulmonary Embolism and hereditary genes
- Effect of using Plavix and Aspirin in Transient Ischemic Attack (TIA) Stroke patients
- Increased Prevalence of Cerebrovascular Accidents (CVA) – Stroke, How can we reduce?
- Risk factors of Cerebral Venous Thrombosis (CVT) & Modified risk factors of Stroke
- Thrombolytic therapy in Acute Stroke
- Anticoagulation agents and Heparin-Induced Thrombocytopenia (HIT)
- Mesenteric Venous Thrombosis
- Epidemiology & management of Stroke patients < 50 years
- Unilateral lower extremity swelling
- Decrease the burden of mobility problems to improve the quality of life
- **Cancer**
- Overall incidence of tumors & Leukemia
- Upper Gastrointestinal (UGI ) cancers; risk factors, prevalence and surgery
- Pancreatic cancer
- Colon cancer; risk factors, prevalence, relationship to internal diseases and management
- New trends in management of Colorectal cancer; Laparoscopic & Surgical
- The biology of Cancer Genetics & Genetic determinants of cancers
- Inhibition of Cancer Stem Cell
- Breast Cancer in young age group
- The association between vitamin D deficiency and Breast Cancer
- Activating efficient PHCCs’ role in early detection of Breast Cancer
- Surveillance for early detection of Breast Cancer and recent managements
- Chest Tumors & Lung Cancer.
- Thyroid Cancer in Saudi Arabia; incidence and risk factors
- Prevalence of Thyroid Cytopathology diseases
- Survey for Cancer screening in females 40 years and above
- Palliative Care for Cancer patients and life of survivals.
- Prevalence of Ovarian Cancer.
- Pathogen Induced GI Cancers
- **Respiratory Diseases**
- Bronchial Asthma & Acute Respiratory Failure and Airway Management
- Study of environmental modification for reduction of Bronchial Asthma Exacerbations
- Adherence of chest physicians to the Stepwise Approach in management of Bronchial Asthma
- The awareness of the proper usage of Inhalers among Asthmatic Patients
- Transfusion-Related Acute Lung Injury (TRALI)
- Chronic Lung diseases
- ‏Pneumothorax
- **Mental Disorders**
- Mental health issues including Dementia & Alzheimer's disease
- Dementia in Saudi Arabia ; types and prevalence
- Anxiety and Depression
- Epidemiology and prevalence of Substance Abuse
- Addiction treatment program; Mid way houses in Canada and USA
- Epidemiology and determinants of mood and psychotic disorders
- **Gastrointestinal Diseases**
- Liver Poisoning
- Epidemiology and prevalence of Acute Pancreatitis & Appendicitis
- Acute Appendicitis in pregnancy and effect of pregnancy on Inflammatory Bowel Disease
- Hepatobiliary Diseases; prevalence, clinical manifestations, diagnosis, management and laparoscopic versus surgical approaches
- Role of Proton Pump Inhibitors in treatment of Diverticulitis
- Bowel Obstruction and comparison of laparoscopic surgery versus Open surgery
- **Autoimmune Disorders and Immune-Mediated Diseases**
- Systemic Lupus Erythematosus (SLE), research from every direction to identify the prevalence, causes, treatments, and means to prevent and cure this life-threatening disease
- Rheumatoid Arthritis; epidemiology and management
- Prevention and early detection of Cerebrovascular Events in patients with Autoimmune Rheumatic Diseases
- Incidence of Stroke in < 50 years patients with Rheumatic Diseases
- Ankylosing Spondylitis (AS)
- Autoimmune diseases
- Celiac Disease
- Multiple Sclerosis (MS) in Saudi Arabia ; reasons why the prevalence is increasing, case control study
- **Nephrology and Renal Diseases**
- Transplant and post-transplant management - Medications comparison and effectiveness
- Effect of Immunosuppressive Therapy in Nephrotic Syndrome
- Prevalence of Chronic Kidney Disease (CKD) and management; outcome improvement
- Study on Acute Renal Failure with special focus on Drug-Induced Nephrotoxicity
- **Hematologic & Genetic Diseases**
- Prevalence of Sickle cell disease (SCD) in Saudi Arabia and modalities of treatment
- Thalassemia & Hemolytic Anemia
- Blood Diseases
- Hereditary Diseases
- **Ophthalmic Diseases and Optometry**
- Saudi National Eye Study: Prevalence and causes of avoidable blindness and vision impairment in Saudi Arabia
- Preventing Visual Loss From Chronic Eye Diseases
- Low Vision; Care & Services
- The social and economic impact of Uncorrected refractive error in Saudi Arabia
- **Orthopedic Disorders**
- SAUDI FRAX; as a Tool to Evaluate Fracture Risk of Patients. It integrates the risks associated with clinical risk factors as well as Bone Mineral Density (BMD) at the Femoral Neck
- Incidence of Osteoporosis (OP) in patients with Chronic Obstructive Pulmonary Disease (COPD) & Inflammatory Bowel Disease (IBD) and probability to prevent or decrease risk of Osteoporotic Fractures
- Ankle Sprain
- **Neurological Disorders**
- Epidemiology of Neuromuscular Disorders and study the responsible genes
- Muscular Dystrophy
- Epilepsy ; prevalence & management
- Parkinson's patients in the Kingdom, and their classification by: gender, age, geographical distribution, stages of the disease
- **Metabolic Disorders**
- Prevalence & Management of Metabolic Diseases in Saudi Arabia
- **Medical Anthropology (Social Science & Medicine)**
- Overall Burden of Diseases; Prevalence , Causes and Risk factors
- Improve the Ministry of Health's strategy to effectively communicate social outreach programs to raise health awareness
- **Toxicity**
- Vancomycin, Paracetamol and other toxicity
- Prevalence of Alcohol toxicity in our community based on ER visits
- Drug Overdose and Abuse of Analgesic medications
- Study of mode of presentation of Scorpion Sting in ER

1. **Communicable Diseases**

- COVID -19 Pandemic and other Outbreak & Epidemics and Pandemics
- Detection of MERS-CoV and preventive measures cost effectiveness
- Study of the most common causes of Acute Respiratory Distress Syndrome (ARDS)
- Role of different ARDS specific interventions modalities and MERS CoV outcomes
- Use of Steroid for management and outcome of Acute Chest Syndrome and Septic Shock
- Extracorporeal Membrane Oxygenation (ECMO) benefit in severe ARDS of (MERS-CoV) ‎patients
- Drug level variation in ECMO Circuit
- Prevalence and management of Pneumonia and Respiratory System Infections in Saudi Arabia
- Burden of most common infections in Saudi Arabia, their management and prevention
- Sepsis; Early identification and treatment
- Tuberculosis (TB) screening for citizens and expatriates working in KSA
- The relation between Human Immunodeficiency Virus ( HIV/AIDS) and Substance Abuse
- HIV drug resistance and mutation associated with resistance
- Prevalence of H. Pylori Gastritis among adults and effectiveness of Triple Therapy in eradication
- Study of the causes of Sepsis & Septic Shock, modalities of prevention and new standards and timing for management
- Medical Virology and Viral Hepatitis.
- Gene studies responsible for Liver Relapse after Hepatitis Infection
- Gastrointestinal Fungal Infection: prevalence clinical presentation and outcomes
- Fever and Cellulitis
- Vaccines for Preventable Diseases and reducing the proportion of acupuncture during vaccination
- Vector Borne Diseases
- Prevention and Control of Malaria and Dengue
- Elimination of Local Transmission of Malaria and Schistosomiasis
- Brucellosis and Prevention of Brucellosis among Livestock Animals

1. **Basic and Therapeutic Nutrition**

- Anemia ; among females from puberty to 40 years & Iron Deficiency Anemia
- Vitamin D Deficiency and related diseases
- Vitamin D Deficiency & Vitamin D Level and its relation to infectious diseases in students
- The relation between Vitamin D and developing Immune Deficiency Disease
- Psychological Consequences of Vitamin D Deficiency
- Tea and Coffee consumption in relation to Vitamin D laboratory results
- Survey for Blood Glucose Screening in Obese Patients
- Prevalence of Overweight, Obesity and Dyslipidemia & Obesity and depression
- Coliform Bacterial Contamination in schools drinking water
- The effect of different Beverages on the growth of Salmonella and Shigella.
- Nutritional versus Caloric Content in School Cafeterias
- Nutritional support & Prevention of Malnutrition problems
- Osteoporosis
- Improving health nutrition care for community members, and patients
- How can Clinical Nutrition improve health outcomes and cut the cost of health services
- Eating Pattern among Saudi families and its effect on children health
- Assessment of nutrition intake and lifestyle of Saudi families
- Medical Nutrition Intervention for obesity
- Oxidative stress in different population
- Health care centers and nutrition & Consultation for Obesity and statistics
- Nutrition for patients with celiac Disease

1. **Holistic Approaches to Health and Wellness, Behavior & lifestyle**

- Determinants of health
- Promote health through decreasing prevalence of communicable and non-communicable diseases
- Social Support Programs and Interventions
- Cigarette Smoking and Psychic Stress Effect on Hypertension, Heart, and Obesity
- knowledge and Community Behavior in dealing with injuries in public places
- Health education, awareness raising and enhanced protection
- Promote healthy lifestyle
- Get fit to run, do run to get fit (reduce obesity )
- Prevalence of physical inactivity and means of overcoming it
- Reduce Tobacco use especially among school aged children and percentage of smoking cessation in KSA

1. **Public Health**

- Assessment of Knowledge, Attitudes, and Practices (K A P) of Chronic Diseases and Cancer.
- Knowledge, Attitudes, and Beliefs about HIV/AIDS and family members living with HIV patients
- Assessment of Awareness about Breast Cancer risk factors and its screening among women
- K A P related to Breast Cancer screening among female health care professionals
- Raising public awareness about the importance of time interval to safe lives when facing emergencies / accidents /Chest pain / trauma
- Knowledge, Attitude and Practices of public towards primary health care services.
- knowledge and Practices about Dementia and Alzheimer's disease care
- Increase community knowledge and Awareness about Allergic Diseases
- Assessment of health risks, to which community members are exposed and ways to prevent them
- Health Illiteracy and Interventions to increase people awareness
- Health education about preventive and therapeutic care, Hand hygiene & Infection control
- Collaboration between Ministry of Health and Ministry of Higher Education to Develop the Health Education Programs
- Public Awareness about the importance of vaccination
- Engage the community and support volunteers’ participation
- Public health at all level with special concern to adolescent Health

1. **Family Medicine & Home Care**

- Burden and Coping Strategies in Caregivers of Stroke Survivors
- The Effect of Home Caregiver Program for Family

1. **Trauma and General Management**

- Road Safety and Road Traffic Accidents (RTA) in Saudi Arabia and Severity of RTA between cities
- Too much traffic and uncontrolled bad driving habits
- Incidence of trauma and its prevention, trauma care program, mass casualty and trauma mortality
- Reprioritizing the ABCs of Trauma Care and implementation of standardized protocols of polytrauma management
- The overload of Road Traffic Accidents on healthcare services and single-center experiences
- Outcome of early approach of RTA patients by critical care specialist
- Head injury, Traumatic brain injury & epidemiology and risk factors of Chronic Subdural Hematoma (CSDH)
- Ocular trauma and cut wound management
- Cervical spine clearance after trauma
- Geriatric Trauma Resuscitation
- Effectiveness of using Morse Fall Risk Assessment and Humpty Dumpty assessment in the prevention of fall risk
- Comparison of complications of Resuscitative Endovascular Balloon Occlusion of the aorta versus emergency department thoracotomy
- Abdominal surgeries
- Non-operative management of Blunt Hepatic Injury: prevalence and outcomes
- Evaluation, management, indications for and complications of laparotomy in patients with Blunt Abdominal Trauma
- Advances in Minimally Invasive Surgery in Trauma
- Trauma and Massive Blood Transfusions and Transfusion Alternatives
- Current situation of voluntary blood donation in Saudi Arabia; why it is important and how to improve it
- Importance of marketing in blood donor recruitment and retention, toward collaboration with other professionally to improve blood donor management
- The role of Computed Tomography (CT) in the workup of trauma

1. **Environmental Health**

- Food safety
- Water pollution
- Air pollution; Burden of diseases and the risk of pulmonary diseases in Saudi Arabia
- Conducting comparative long-term (prospective cohort) studies on residential areas, that includes varied community strata, to clarify the relationship between environmental risks; respiratory diseases, diabetes, heart and cancer

1. **Pilgrims’ Health**

- Health promotion in Haj
- Prevalence of gastroenteritis, influenza & other infectious diseases among pilgrims and methods of prevention
- Chemical and microbiological analysis of food provided to pilgrims.
- Haj emergency

1. **Women’s Health**
2. **Clinical Obstetrics**

- **Pregnancy Abnormalities and Abnormal Pregnancy**
- Gestational Diabetes (GDM) and medical interventions
- Pregnancy-Induced Hypertension (PIH)
- Thromboembolic disorders during pregnancy
- Bronchial Asthma during pregnancy
- Pregnancy and Autoimmune diseases
- Prevalence of Hypertension , DM, VTE & Thyroid disorders in pregnancy and pregnancy outcome
- Sickle Cell Disease in pregnancy
- Haematological diseases during pregnancy
- Malaria infection during pregnancy
- Prevalence of Malnutrition; Obesity & anemia among pregnant women
- Vitamin D during pregnancy
- Impaired Glucose Tolerance (IGT) during pregnancy
- Preeclampsia & prevalence
- Preeclampsia, Eclampsia, and HELLP syndrome
- Association of Calcium supplementation during pregnancy and reduction of the risk of Preeclampsia
- First Trimester Ultrasound
- Uterine and Umbilical Artery Doppler ultrasound
- Management of high-risk pregnancy
- Placenta Accreta spectrum: Risk factors, diagnosis and management
- Placenta Previa; causes, prevention & Management of intraoperative heamorrhage
- Modern aspects of uterine bleeding treatment with placenta previa
- Association of placenta previa with repeat cesarean section
- Advanced maternal age and abnormal pregnancy
- Ectopic pregnancy & Incidence rate and prevalence in primigravida
- Antepartum Haemorrhage (APH)
- Sudden intrauterine fetal death (IUFD)
- Multiparity
- **Miscarriage**
- Recurrent Miscarriage (RM) and Clexane
- Recurrent Miscarriage and Autoimmune diseases
- Medical management of Miscarriage & Post-Abortion Care (PAC)
- Comparative studies between surgical and medical procedures for induction of abortion for non-viable pregnancies
- **Difficult Deliveries**
- Maternal Mortality
- Influence of medico-legal issues on Obstetricians’ decision-making and impact on patients
- Caesarean Section (CS) determinants & Reducing rates
- Vaginal Birth After C-Section (VBAC) ; success rates and outcomes
- The use of Oxytocin during labor
- Postpartum complications & management
- Improving intrauterine fetal monitoring methods to avoid large number of caesarean sections
- Vaginal birth perineal incision
- Impact of mode of delivery on pelvic morphology: using translabial 3D ultrasound
- Maternal and fetal outcomes in term premature rupture of membrane
- **Advances in Diagnostic Equipment and Techniques**
- Anomaly Scan
- Genetic Testing
- Antenatal screening for Down Syndrome for creating Database & National Down Syndrome Cytogenetic Registry
- **Fetal Growth Retardation and Abnormal Development**
- Consanguineous marriage and incidence of congenital anomalies in Saudi Arabia
- Breastfeeding future

1. **Gynecologic Oncology, Urogynecology and Minimally Invasive Gynecologic Surgery**

- **Gynecologic Oncology**
- Effectiveness of colposcopy in cervical screening
- Screening for early detection of cervical cancer
- Role of outpatient endometrial biopsy in endometrial cancer screening
- Early detection of breast cancer and relation to cervical cancer
- Immune response to fight cancer
- Women compliance with the Annual gynecologic exams for preventive health screening and education
- **Urogynecology**
- Urinary incontinence in postmenopausal women – causes, symptoms and treatment
- **Minimally Invasive Gynecologic Surgery**
- Domestic violence
- Correlation between postoperative wound infections with hemoglobin levels
- Perioperative Thromboprophylaxis

1. **Reproductive Endocrinology and Infertility**

- **Reproductive Endocrinology**
- Women’s health promotion
- Polycystic Ovary Syndrome (PCOS); Prevalence, causes & management
- Prevalence of polycystic ovary syndrome in adolescents
- Knowledge about irregular menstrual cycle among females
- Hormone Replacement Therapy (HRT)
- Management of endometrial hyperplasia in premenopausal age
- Preconception Screening & Education
- **Infertility**
- Common causes of infertility
- Increasing In Vitro Fertilization (IVF) success Rate
- In Vitro Fertilisation (IVF) & advanced techniques

1. **Familial Diseases**

- Premarital screening program and other preventive Genetic programs
- Prenatal genetic screening and diagnosis

1. **Child and Neonatal Health**
2. **Newborn Health**

- Neonatal mortality and its causes
- Complications of neonatal transport from peripheral hospitals
- Causes & follow up of Prematurity in Saudi Arabia
- The chances of survival for premature babies & the risk of premature babies having severe disabilities
- Introduction of Respiratory syncytial virus (RSV) Vaccine to the immunization schedule
- Newborn health care services development
- Neonatal Screening program
- Interventions to improve preterm birth outcomes

1. **Nutrition, Behavior & Lifestyle**

- Neonatal and child growth and Failure To Thrive (FTT)
- Development of specific “ Growth Chart Percentile” for each Arabic country
- “Growth Reference Study” Development of Growth parameters according to local data
- Effect of healthy food on school performance
- Factors associated with vitamin D deficiency
- Breastfeeding ; Rate in Saudi population, awareness and factors promoting breastfeeding for > 6 months
- Anemia is a common problem and its effect on school age children
- Nutritional problems in young children and prevention programs
- Overweight and obesity among Saudi children
- Anemia and rickets

1. **Respiratory Diseases**

- Pediatric respiratory problems
- Bronchial Asthma & its prevalence in KSA
- How different factors may modulate adherence to asthma treatment and the importance of increasing adherence to asthma therapy
- Home environment of children with bronchial asthma

1. **Infectious Diseases**

- Respiratory Distress in young children, is it Asthma?
- Prevalence of Respiratory Distress Syndrome (RDS) in Saudi Arabia
- Excessive antibiotic use for acute respiratory infections
- Neonatal Intensive Care Units (NICUs) infection rates & Respiratory support
- Pneumonia among 5 months to 2 years children
- Bronchiolitis & bronchiolitis management
- The relation between passive smoking and Upper Respiratory Tract Infections (URTI) in children
- Fever and Pediatric Infectious Diseases
- Brucellosis needs supportive action from various sectors, including those responsible for food safety and consumer education
- Epidemiology & management of Meningitis
- Prevalence of TB among children
- Hand-foot-and-mouth disease (HFMD)

1. **Genetic & Hematologic Diseases**

- Prevalence of genetic diseases among children in KSA and Stem Cell Therapies
- Sex Linked surgical diseases at southern KSA
- The landscape of genetic diseases in Saudi Arabia; prevention, diagnosis and management
- Sickle Cell Disease; Anemia and Infection
- School performance of children with Sickle Cell Disease
- Blood diseases such as Thalassemia
- Syndromes as Down Syndrome (DS)
- Complications of chronic hemolytic anemia
- ABO incompatibility

1. **Endocrine Diseases**

- Diabetes Miletus type 1 and Diabetic ketoacidosis (DKA)
- Risk factors for Insulin-Dependent Diabetes Mellitus (IDDM) in Saudi children
- Quality of life and depression in diabetic children
- Thyroid disorders

1. **Developmental Anomalies & Disabilities and Management**

- Autism
- Diseases and learning difficulties
- Attention Deficit Hyperactivity Disorder (ADHD); Genetic & Environmental risk factors and Prevalence
- What is the prevalence of ADHD among individuals with substance use disorders SUDs and what is the impact on the risk for SUDs
- The Clinical and Cost effectiveness and optimum length of brief group based ADHD focused parent training interventions
- The validity of standardized verbal and nonverbal IQ tests in the Saudi population and the impact of cultural linguistic and behavioral differences
- The quality assessment of Translation , Adaptation and Validation of DSM-5 and ICD11 based assessment tools for screening clinical assessment
- Congenital myopathy & Congenital anomalies in the kingdom
- Effect of children exposure to electronic devices on emotional, behavioural response difficulties and Autism

1. **Genitourinary Diseases**

- Recurrent Urinary Tract Infections (RUTI)
- Urinary Tract Infection (UTI) and Extended Spectrum β-Lactamase (ESBL)-producing bacteria
- Hypospadias and testicular problems in children
- Awareness about acute scrotum approach among general practitioners and ER doctors
- Neurogenic bladder and nocturnal enuresis
- Family awareness regarding Nephrotic syndrome patients

1. **General Management Approaches and Interventions**

- Acute abdomen and pediatric surgery
- Hirschsprung's disease cohort study - Anorectal malformation study of quality of life in operated patients
- Acceptance of parents and their knowledge to deal with the conversion of stool through the abdomen
- Awareness of the importance of kidney donation for patients with renal impairment
- Congenital defects and kidney malformations
- Epidemiology and management of congenital anomalies including hernias
- Prevalence of acute appendicitis in children in Saudi Arabia
- Surgical management of chest wall deformities
- laparoscopic versus open surgery in children

1. **Pediatric Emergencies**

- Emergency, children abuse, anaphylaxis in children
- Epidemiology of Accidental poisoning in children
- The use point-of-care ultrasound in the Neonatal Intensive Care Unit (NICU)
- Neonatal Pediatric resuscitations and transportation
- Pediatric ICU mechanical ventilation ; new modes and strategies
- Resuscitation timing and Cerebral Palsy (CP)

1. **Neurological Diseases**

- Neurodegenerative and Genetic Neurological diseases
- Epilepsy
- Quadriplegia; causes and prevention

1. **Gastrointestinal Diseases**

- Necrotizing Enterocolitis (NEC)
- prevalence of Basidiobolomycosis infection among children in the southern Aseer region
- Fecal Incontinence
- Causes of GIT problems among children in KSA
- Diarrheal diseases in pediatric

1. **Cardiovascular Diseases**

- Congenital and pediatric heart diseases in Saudi Arabia
- Persistent Pulmonary Hypertension of the Newborn (PPHN)

1. **Trauma and General Management**

- Prevalence and consequences of pediatric trauma including their management and the best way for prevention
- Epidemiology of Road Traffic Accident (RTA) injuries among children
- The outcome of brain protective strategy in children involved in Road Traffic Accidents (RTA)

1. **Dermatological Diseases**

- The association between Vitamin D deficiency and skin diseases
- Association between Eczema and Bronchial Asthma with vitamin D deficiency

1. **Metabolic Diseases**

- Causes of Metabolic diseases
- Newborn screening for early detection and management of many congenital genetic and metabolic disorders

1. **Pediatric Tumors**

- Prevalence and management of pediatric tumors
- Skull Vault Hemangiomas (SVH), as a benign slow-growing vascular neoplasms

1. **Ophthalmic Diseases**

- Retinopathy Of Prematurity (ROP) in Saudi Arabia
- Incidence of Retinopathy Of Prematurity (ROP) in Gestational Age (GA) less than 30 weeks or weight less than one kilogram

1. **School Health**

- Vaccinations
- Health education about preventive care, hand hygiene & infection control.
- Chronic diseases prevention programs for school aged children
- Kuwait preventive school program
- School Health Program (Nutrition and cantinas) in Fenland, School Health Program (Physical activity in UK) and Health promotion school in UK England

1. **Immunological Disorders**

- Immunodeficiency Diseases in Saudi Arabia
- Acute Rheumatic Fever and Autoimmune diseases

1. **Geriatric Health**

- Diseases of digestive system in the elderly
- Geriatric diseases and problems

1. **Biomedical Technology Related Fields & Devices**

**1. Molecular Biology and Genetics**

- Molecular biology and epidemiology
- Genetic quality & Epigenetics
- Mapping genes importance towards the development of new biochemical treatments
- Personalized Diagnostics
- Cellular and molecular functions of the latest medications for the treatment of high blood pressure
- Availability of metabolic disorders testing

**2. Immunobiology**

- Investigation of anti-cancer activities of extract from Momordica charantia on gastric cell carcinoma in Vitro
- Immunology

**3. Medical Laboratory**

- Methods of manufacturing stem cells and keeping them in the laboratory and study their effect on the treatment of serious diseases
- Identifying the food that can be consumed Pre-analysis (food labels & nutrition facts)
- Analysis of popular and local meals and assessment of the nutritional status of the Saudi population
- Apoptosis and chronic liver disease
- Quality, safety & infection control measures in laboratories
- Cost effectiveness of routine investigation at central hospitals
- The accuracy of the readings of the devices -Cost and quality
- Comparison between blood film malaria and strip method
- Rehabilitation of "Phlebotomist" blood withdrawal specialists to avoid damage samples
- Diabetes and Factor deficiency
- Effects of prolonged exposure to dab detection reagent
- Preanalytical and analytical errors in the measurement of Adrenocorticotropic hormone (ACTH) levels

**4. Hematology**

- Evaluation of hemoglobin and MCV in-patient with dental caries
- Incidence of platelets refractoriness
- Blood banks & Blood transfusion and safety
- The importance of stem cells in blood transfusion medicine
- Extended phenotyping of red blood cells for blood donors in KSA
- Unifying patient file in the health sector to reduce blood transfusion risk
- Current situation of voluntary blood donation in Saudi Arabia; why it is important and how to improve it
- Post blood transfusion reaction, ABO discrepancies & risks of blood transfusion and their prevention
- Effect of “old” versus “fresh” transfused red blood cells on patients

**5. Biomedicine & Pathology**

- Frequency of antibody formation in oncology patients
- Taken biopsy for cancer cells
- Molecular Pathophysiology of Cancer Development
- Fibromatosis
- Primary Diffuse B-Cell Thyroid Lymphoma
- Evaluation of mammaglobin as a breast tumor marker for early diagnosis
- Ways of reducing waste in solutions and laboratory reagents
- Flow cytometry

1. **Medical Radiology & Devices**
2. **Breast Imaging**

- Prevention of Breast Cancer by use of Mammograms & Breast Cancer monitoring
- Personalized Breast Screening and Breast Density

1. **Cardiovascular Radiology**

- Advanced vascular ultrasound
- Cardiac radiation

1. **Emergency Radiology**

- Emergency diagnosis

1. **Head and Neck Radiology**

- The most common tools for diagnosis of Thyroid Gland Diseases

1. **Musculoskeletal Radiology**

- The diagnostic role of bone scan in distinguishing between Acute Osteomyelitis and Bone Infarction

1. **Neuroradiology**

- Advantages and disadvantages of Glomerular Filtration Rate diagnostics in Nuclear Medicine
- The most accurate modality to detect Parkinson's Disease

1. **Nuclear Radiology**

- Facts of body weight, height, and body mass index on thyroid volume among healthy Saudi males using nuclear imaging
- The sensitivity of Magnetic resonance imaging (MRI) for the detection of Multiple Sclerosis (MS) lesions

1. **Radiation Oncology**

- The role of Magnetic Resonance (MR) imaging in oncology

1. **Health Service Delivery**

- Artificial Intelligence in medical imaging diagnostics
- Quality and safety in radiation dose; specially in pediatric patients
- The level of education and training in radiology department (RD)
- Frequent MRI requests
- The evolution and benefits of advanced clinical radiology
- Radiographic image quality
- Radiation Safety & protection from radiation exposure
- A computerized Tomography (CT) Scan & Reduction of patients CT doses
- Patient education about radiation
- Measuring the extent of knowledge about radiation protection protocols among workers in RD
- Ultrasound scanning technique
- Radiation risk and safety among healthcare practitioners
- The importance of education in radiation safety in MRI Imaging
- The Accuracy of Radixact Treatment Delivery System

1. **Dental Health**
2. **Assessment of Dental Problems’ Burden**

- Early Childhood Dental Caries: risk factors , Prevalence and Treatment outcomes
- Behavior management of Pediatric Dental Patients
- Prevalence of dental health problems in Saudi population and Prevention
- Prevalence of diagnosed Temporomandibular disorders among Saudi Arabian children and adolescents
- Incidence and prevalence of C-shaped Canal in Saudi population
- Prevalence of Hypodontia
- Impact of Fluoride on dental health quality
- Risk factors for tooth loss in adults
- Impact of marketing cosmetic treatment on oral health
- Oral Health Status; community data
- Prevalence and trends of oral diseases

1. **Exploring knowledge, Attitude, and Practices**

- Awareness and practice of tooth brushing techniques and proper oral hygiene
- Studying the ways to increase community awareness about ;
- Genetic diseases related to dental health
- Gum complications in diabetics
- Fluoride's Importance to teeth
- Dental floss
- Oral health knowledge, attitude and practices among public and health professionals
- Implementation of preventative measures
- Patients' knowledge and awareness of Dental Implants ; Care & Instructions to be followed after Dental Implant Surgery
- Awareness and Acceptance of Dental Implants as a Treatment Modality for Replacement of Missing Teeth among Patients
- Perception and Awareness of Prosthodontic Rehabilitation in Saudi Arabia
- Periodontal disease ; Education, Awareness and knowledge among community

1. **Dental Hygiene Education for ; Workforce and Public**

- Electric vs. manual tooth brush
- Water Fluoridation: dental and other human health outcomes
- Radiation protection of ; staff in dental radiology and patients
- Effect of motives for food choice on oral health
- Impact of medical education on service level
- Causes of gingivitis

1. **Healthcare & Hospital Administration**

- Future MOH Plans for dental caries control
- Establishment of Integrated oral health Centers
- Meeting the challenges of special needs dentistry
- Access to preventive oral health care and its impact
- The role of primary health care centers in reducing prevalence of dental caries in permanent teeth
- Antibiotics ; guidelines, resistance and rational use
- Career satisfaction among dental practitioners
- Prevalence of neck and back pain among dentists
- Impact of electronic appointment on patients satisfaction in dental clinics
- Infection control in dental clinics
- Evaluation of quality of public dental health service from different dimensions
- School-Based Dental Programs; Prevent Cavities and are cost-effective
- Dental malpractice

1. **Dental Problems Management**

- Endodontics & Role of interceptive orthodontics in early mixed dentition
- Fluorosis
- Cleft lip and cleft palate
- Therapeutic plans
- Comparative evaluation of the efficiency of cordless versus cord techniques of gingival retraction
- Restored vitality of teeth &Dental implants
- Management of geriatric oral problems with comorbid conditions and physiologic changes
- Dental management in the medically compromised patients with; renal, cardiovascular , hematologic, DM and other diseases
- Periimplantitis around dental implants in smokers and non-smokers
- Oral health status and treatment needs among pregnant women
- Class III malocclusion and the effect of early timely treatment
- Comparison of the marginal bone loss in one-stage versus two-stage implant surgery
- Dental amalgams—boon or bane?
- Influence of orthodontic treatment on root resorption
- The mechanism of ending cases in the laboratory
- Selection of Biomaterials for Dental Applications
- Effectiveness and biocompatibility of dental management biomaterials
- Advanced Instruments in dental care services
- Problems related to polymerization Shrinkage and to provide clinicians the opportunity to improve the quality of their restorations
- Polymerization Shrinkage Stress: current techniques for posterior direct resin restorations
- Gingival microleakage of class II bulk-fill composite resin restorations
- Ideal restoration for deep cavity and subgingival area
- Availability of restorations in dental field
- Treatment Modalities Regarding management of Open Apex In young age
- Stem Cells & Dental Stem Cell regeneration techniques may spell the end of tooth decay
- The importance of implantation of pit and fissure sealant

1. **Oral and Maxillofacial Surgery**

- Oncology
- Fractures of the face and jaws & Effect of accidents
- Orthopedic-Plastic surgery of the face and jaws
- Bone grafts in dentistry ; success rate and complications

1. **Medications**
2. **General Pharmaceutical Practices**

- **Medication Safety**
- Pharmacist's Role in Medication Safety
- Impact of medication errors on provided health care services
- Reduction of medication prescribing errors
- Medical errors as a leading cause of death
- Adverse drug reactions
- Effect of antiepileptic drug therapy on thyroid hormones
- Analysis and evaluation of prescriptions in KSA hospital pharmacies
- Pharmacists' interventions in the management of patients with chronic kidney disease
- Optimization of Antidepressant use
- Ambulatory care
- Impact of clinical pharmacist in intensive care unit & infections control
- Policy for reducing the risk of Venous Thromboembolism (VTE) implementation
- Rational use of medicines
- Medication guide for genetic diseases
- Practical ways to improve patients adherence to prescribed medication
- Safety and effectiveness of Complementary and alternative medicine (CAM)
- **Medications Action & Interactions**
- ICU management ; Ventilator dependence, Sedation, Fluid responsiveness and evaluation
- Preparation and Evaluation of Theophylline and assessment of the effect on lung function
- Evaluation of Parenteral Nutritional (PN) support in the surgical and medical wards
- Effect of Antidepressant drug use on electrolytes blood sugar levels
- Practical approach to managing Dyslipidemia: Role of Statins
- Pharmacokinetics of drugs in humans; Effect of Obesity
- Intravenous Solutions - Dosing unit - Automated drainage for chronic disease patients
- Antiplatelet drug interactions with Proton Pump Inhibitors
- Drug-drug interactions
- Drug-food interactions
- Drug Alertness
- Psychotropic Medications
- **Antimicrobials**
- Impact of Antimicrobial Stewardship Programs application on healthcare associated infections, patient safety, costs control, and antimicrobials resistance
- A surveillance study of prevalence and antimicrobial resistance pattern of microbes isolated from different hospital sections
- Rational use of antibiotics in general surgery
- Antibiotics utilization at all levels & Exploring the common features of antibiotic therapy misuse
- Bacterial Culture and Antibiotic Susceptibility Testing ; lower risks of complications and antibiotic resistance
- Effect of antibiotics on HIV
- Antibiotics side effects
- Multidrug resistance; in treatment of TB
- Impact of the length of stay in ICU in prevalence of Multidrug-Resistant Organisms (MDROs)
- **Computerized Chronic Disease Management System**
- Impact of automated solution on providing higher quality patient care, workload and dispensing errors in a hospital pharmacy
- Medication Refills from the nearest hospital
- Drug Delivery
- Electronic Recipe; impact and challenges

1. **Pharmaceutical Policy and Hospital Administration**

- Health Policy and Regulation
- Application of national or international dose guidelines in ED will improve the outcome in dollars , improve bed management & help to decrease the staff assault or abuse
- In-depth assessment of the supply management system of essential medicines, disbursement and stock availability and shortage
- Management of Medication Exchange and Sharing Network Program (MESNP) initiative to cope with drug shortages in MoH hospitals
- Emergency Supply Prescription approaches and Inhalation therapy
- IV rooms
- Pharmacovigilance: ensuring the safe use of medicines
- Supply Chain Management (SCM) application impact
- Impact of Drug Information Systems in protecting patients from drug interactions
- Barriers to Medication Errors Reporting
- Knowledge, attitudes & practices of healthcare professionals in hospitals towards the reporting of adverse drug reactions

1. **Digital Transformation of Medicine; Pharmaceutical Technology**

- Performance improvement of Computerized Provider Order Entry (CPOE), as the best exemplified digital transformation of medicine
- The Computer-Based Unified Patient Record; as the principal repository for patient's information to enhance the quality, effectiveness and efficiency of health care delivery.
- Employing routine and innovative forms of Information and Communications Technology (ICT) to address health needs, By linking patients' files with mobile applications (mHealth)
- Pharmacy Informatics involves working with new technology, Automation and Information systems
- Examining the role of new technology in pharmacy: now and in the future
- Outpatient Pharmacy Automation System
- Electronic Prescribing Service (EPS) Usability: Reduction of workload and prescribing errors
- Telehealth: Technology meets health care

1. **Pharmaceutical Industry ; Drug Innovation, Quality, Safety and Security**

- Study of challenges, limitations and applications of Pharmacoeconomics by comparing costs and consequences of pharmaceutical products and services
- Activate and provide medical and non-medical products in all KSA cities
- Value based concept with regard to expensive medication in MOH
- Application of evaluation methods of pharmaceutical products and services in terms of; cost minimization analysis, cost benefit analysis, cost utility analysis and cost effectiveness analysis
- Evaluation of factors affecting drug quality and impact on patient safety and health
- Development of; Blood Thinner medications, Diabetes medications, Dermal drugs and Chemotherapeutic agents
- Development of; drugs for Liver diseases and Vaccines
- Recent advances in extraction of Antioxidants from plant by-products processing industries
- Knowledge, beliefs and behaviours regarding the adverse effects of medicines
- Development of new antibiotics to overcome accelerated emergence of antibiotic-resistant pathogens and combating the more scientific barriers to drug discovery
- New treatment for Osteoarthritis
- New Oral Hypoglycemic Agents
- New Antiepileptic Drugs  (AEDs)  Compared to traditional AEDs
- Multiple Sclerosis (MS) disease-modifying therapies research update
- Unit dose
- Animal Test
- Development of drugs for Chronic diseases
- Radiopharmaceuticals

1. **Self-Medication Practices and Alternative Medicine**

- Evaluation of self-medication practices with herbal medicines and modern pharmaceuticals without prescription ; patterns, contributing factors and health effects
- Use of non-narcotic, non-prescription analgesics to control pain and inflammation
- Use of alternative medicine (plants and herbs) to fight against germs

1. **Pharmaceutical Education**

- The provision of Drug Information (DI) to patients, caregivers, and health care professionals
- Pharmaceutical education in patient counseling
- Drug monitoring , Patient compliance , Medication adherence
- Medication safety, Adverse drug reactions, Drug interaction issues and Hazardous medication
- Educating Patients about missed medication doses
- Educating Diabetics and patients with Inherited Blood Disorders
- Inhaler technique education by a clinical pharmacist and Asthma & Chronic Obstructive Pulmonary Disease (COPD) control among patients
- Health Education about;
- Smoking cessation
- Eye diseases
- Infectious diseases
- Genetic Hematology
- Anemia
- Chronic diseases
- Rheumatic disorders

1. **General Management and Innovative Approaches and Interventions**

- Stem cell-based regenerative medicine
- Laparoscopic versus Open Surgery
- laparoscopic surgery in obese patients
- Early versus Delayed Surgical Repair or Referral and clinical outcomes
- Comparison of conservative and operative management
- Effectiveness of Hyperbaric Oxygen Therapy (HBOT) for wound healing, Carbon Monoxide Toxicity and Autism Spectrum Disorder (ASD)
- Sepsis; Early identification and treatment
- Postoperative respiratory impairment contributes to both surgical and anesthetic risk
- Minimally invasive surgical approaches versus open procedures
- Prevalence, microbiology and care of Surgical Site Infection (SSI)
- Incidence of postoperative complications among day surgery cases
- Complications of laparoscopic surgery and patient Safety in general Surgery
- Cost-effectiveness studies; that assess Interventional Techniques in general surgery
- Laparoscopic Hernia Repair
- The shift of an intestinal “microbiome” to a “pathobiome” governs the course and outcome of sepsis following surgical injury
- Bariatric Surgery:
- laparoscopic techniques in Bariatric surgery
- Bariatric surgery current & future
- Complications of Bariatric surgery
- Portomesenteric Vein Thrombosis after Laparoscopic Sleeve Gastrectomy: incidence, risk factors and management single center experiences
- The Impact of Bariatric Surgery on patient health and Type 2 Diabetes Mellitus

1. **Emergency Management (Critical Care Medicine)**

- Guideline recommendations for the diagnosis, treatment, and follow-up of patients with Acute Anaphylaxis
- Acute & chronic pain management in emergency (ER) medicine in Saudi Arabia
- Study of common Intensive Care Unit (ICU) infections & Prevalence of antibiotics resistance
- Study of the risk of acquisition of Infectious disease during Emergency room visits
- Post Sleeve Gastrectomy: Early complications presenting in ER prevention and management
- Convulsions
- Surgical operations emergencies
- Ultrasound applications in EM
- Effectiveness of the i-STAT blood analyser in rapid decision-making in the Emergency Department
- STEMI Management in ER
- N-Acetylcysteine in Acute Liver Failure (ALF)
- Delirium: Causes and management of delirium in Intensive care unit patients
- Hemodynamics; monitoring & management
- Food related emergencies , including Glucose-6-Phosphate Dehydrogenase (G6PD) Deficiency
- Central consultation by qualified physicians will improve outcome
- Screening and observing daily ER cases and records prospective
- Pre Referral care and patient Transfer in ICU
- Public health emergency preference

1. Physical Therapy & Rehabilitation
   1. **Sports Injury**

- Effectiveness of using taping technique in sport injury cases
- Collaborative research with Saudi Physical therapy associations , Saudi Federation for sports medicine and Medical committee for Saudi football federation
  1. **Neurology**
- Neurorehabilitation
- Spinal cord injury and physical therapy
- Effectiveness of manual therapy for management of patients with migraine and nonspecific low back pain
- Multiple Sclerosis
- Effectiveness of Trigger Point Dry Needling for treatment of chronic pain
- Impact of electrical therapy after stroke
- Effectiveness of electrical stimulation of peripheral nerve injury
  1. **Orthopedics**
- Orthopedic & musculoskeletal rehabilitation
- Comparing electric treatment, therapeutic exercise and lower back muscles elongation on treating lower back chronic pain
- Awareness, Knowledge and Belief of physical therapists practitioners about vestibular rehabilitation
  1. **Physical Therapy Specialties in General**
- Increase awareness among patients about the role of physiotherapists
- Congenital Diaphragmatic Hernia (CDH) postoperative protocol
- Aerobic exercise and its impact on the reduction of chronic diseases

**Agenda: 3. National and International Collaborative Research Topics Options of Headquarters’ Leaders**

| **Participant No.(34)** | **Research Topics** |
| --- | --- |
| 1 | Addiction program Mid way houses in Canada and USA |
| 2 | Antibiotics utilization at all level & Public health emergency preference and Haj |
| 3 | Apply the insurance for all individuals in KSA similar to the UAE |
| 4 | artificial intelligence |
| 5 | collaboration between ministry of health and British agency of Care Quality Commission to come out with regulations for plastic surgery |
| 6 | Collaboration between Ministry of Health and Ministry of Higher Education to Develop the Health Education Programs |
| 7 | Cost effectiveness analysis of the national screening program for breast cancer in KSA |
| 8 | Diabetes interventions |
| 9 | Disease prevention and control |
| 10 | Economic impact in health system |
| 11 | Emerging infectious diseases & Preparedness for and response to public health emergency |
| 12 | Increase the knowledge and awareness among the community about allergies and Enhance the Autism Centers in Saudi Arabia |
| 13 | Evaluation of economic impact and analysis of complementary medicine approaches, as a step for integration into the main healthcare system |
| 14 | Health care access |
| 15 | Health Statistics in collaboration with General Authority for Statistics and Bio indicator |

Continue table……

| **Participant No.(34)** | **Research Topics** |
| --- | --- |
| 16 | Health technology system in Saudi Arabia |
| 17 | Home TPN(total parenteral nutrition) , BMI (body mass index), Car T Cell Therapy |
| 18 | Kuwait preventive school program |
| 19 | Measuring the impact of health campaigns and Measuring the impact of MOH campaigns among the community during 2018 |
| 21 | Plain packing with Austrian campaign and illicit trade in tobacco products among the gulf region |
| 22 | Policy to sale healthy food with no TAX added |
| 23 | Population health and economic cost accountable of model |
| 24 | Prevention and control of Malaria and Dengue |
| 25 | Rate of breastfeeding in Saudi population & Analysis of popular and local meals and Assessment of the nutritional status of the Saudi population |
| 26 | Promote and share health information |
| 27 | Public health in all policy |
| 28 | public health laboratory , intervention to address the non-communicable disease and ethic recreation of digital health |
| 29 | Saudi Physical therapy associations , Saudi federation for sports medicine and Medical committee for Saudi football federation |
| 30 | School Health Program (Nutrition and canteens) in Fenland, School Health Program (Physical activity in UK) and Health promotion school in UK |
| 31 | shared health |
| 32 | Spiritual support program in Stamford in the USA |
| 33 | Track of Counterfeit Medication. |
| 34 | viral vaccine , cancer overall and geriatric patients |

# **References:**

1. Remme JHF, Adam T, Becerra-Posada F, D’Arcangues C, Devlin M, Gardner C, et al. Defining research to improve health systems. PLoS Med. 2010;7(11).

2. Don de Savigny, Harun Kasale, Conrad Mbuya and GR. Fixing Health Systems. 2nd Editio. the International Development Research Centre PO Box 8500, Ottawa, ON, Canada K1G 3H9 www.idrc.ca; 2008.

3. Ministry of Health. Transformation Strategy. Model Archit Pract. 2017;265–7.

4. Commonwealth Secretariat. A Systems Framework for Healthy Policy Advancing, Global Health Security and Sustainable Well-being for All. the Commonwealth Secretariat. 2016.

5. World Health Organization. Improving the quality of health services - Tools and Resources. WHO Service Delivery and Safety Department. 2018. 1–59 p.

6. Rockville M. Health Systems Research. [Internet]. Health Systems Research. Agency for Healthcare Research and Quality. 2020. Available from: https://www.ahrq.gov/healthsystemsresearch/index.html

7. WHO. Monitoring the Building Blocks of Health Systems : a Handbook of Indicators and. 2010;110.

8. World Health Organization. A strategic framework for health workforce development in the Eastern Mediterranean Region. East Mediterr Health J. 2017;23(5):388–9.

9. Monday hris B on. What is a Health Information System? [Internet]. Data inside Digital Guardian’s Blog. 2020. Available from: https://digitalguardian.com/blog/what-health-information-system

10. Rouse MSW and AD. Health Information Technology Office for Civil Rights Headquarters U.S. Department of Health & Human Services [Internet]. TechTarget 20. 2019. Available from: https://searchhealthit.techtarget.com/definition/Health-IT-information-technology

11. Commision E. European Commision. Eur Commision [Internet]. 2017;(2012):28–32. Available from: https://ec.europa.eu/health/human-use/falsified_medicines_en%5Cnhttps://ec.europa.eu/health/sites/health/files/files/eudralex/vol-1/reg_2016_161/reg_2016_161_en.pdf,

12. Barbabella F, Melchiorre MG, Papa R, Lamura G. How can eHealth improve care for people with multimorbidity in Europe? Health Systems and Policy Analysis. 2016;

13. Kazi DS. From Innovation to Implementation. J Am Coll Cardiol. 2016;64(24):2616–8.

14. Halabi SF. Access to Medicines and Vaccines. Intellect Prop New Int Econ Order. 2018;(April):91–104.

15. Government of Ireland. Research Priority Areas 2018 to 2023. Innov 2020 [Internet]. 2018;26. Available from: https://dbei.gov.ie/en/Publications/Publication-files/Research-Priority-Areas-2018-to-2023.pdf

16. WHO. Health financing [Internet]. WHO. 2020. Available from: https://www.who.int/health-topics/health-financing#tab=tab_1

17. WHO. Cost effectiveness and strategic planning (WHO-CHOICE) [Internet]. 2020. Available from: https://www.who.int/choice/en/

18. WHO. Health system governance [Internet]. WHO. 2020. Available from: https://www.who.int/health-topics/health-systems-governance#tab=tab_1

19. WHO. Health Emergency and Disaster Risk Management: Overview [Internet]. Health Emergency and Disaster Risk Management Fact Sheets. 2019. 48 p. Available from: https://www.who.int/hac/techguidance/preparedness/health-emergency-and-disaster-risk-management-framework-eng.pdf?ua=1

20. World Health Organization; World Health Assembly Update, 25 May 2019 [Internet]. World Health. 2019. Available from: https://www.who.int/news-room/detail/25-05-2019-world-health-assembly-update

21. World OH. Co V I D ‑ 19 Strategy Up Date. 2020;(April):18.

22. Health M of. Health Sector Transformation Strategy [Internet]. 2017. Available from: https://www.moh.gov.sa/en/Ministry/vro/Documents/Healthcare-Transformation-Strategy.pdf

23. Memish ZA, Jaber S, Mokdad AH, AlMazroa MA, Murray CJL, Al Rabeeah AA, et al. Burden of disease, injuries, and risk factors in the Kingdom of Saudi Arabia, 1990-2010. Prev Chronic Dis [Internet]. 2014;11:E169. Available from: http://www.ncbi.nlm.nih.gov/pubmed/25275806%5Cnhttp://www.pubmedcentral.nih.gov/articlerender.fcgi?artid=PMC4184091

24. Khalil MKM. Integrative Medicine: The Imperative for Health Justice in the Other Side of the World. Vol. 24, Journal of Alternative and Complementary Medicine. 2018.

25. Arena R, Lavie CJ, Hivert MF, Williams MA, Briggs PD, Guazzi M. Who will deliver comprehensive healthy lifestyle interventions to combat non-communicable disease? Introducing the healthy lifestyle practitioner discipline. Vol. 14, Expert Review of Cardiovascular Therapy. 2016.

26. Weeks J. The Berlin Agreement: Self-Responsibility and Social Action in Practicing and Fostering Integrative Medicine and Health Globally. J Altern Complement Med. 2017;23(5):320–1.

27. Khalil MKM, Al-Eidi S, Al-Qaed M, AlSanad S. The future of integrative health and medicine in Saudi Arabia. Integr Med Res. 2018;7(4).

28. Harvard T.H. Chan School of Public Health 2020.The Nutrition Source. Disease Prevention [Internet]. Harvard T.H. Chan School of Public Health 2020. 2020. Available from: https://www.hsph.harvard.edu/nutritionsource/disease-prevention/

29. WHO. Briefing of Member States and partners on the double burden of malnutrition [Internet]. World Health Organisation. 2019. Available from: https://www.who.int/news-room/events/detail/2019/12/18/default-calendar/geneva-double-duty-actions-to-address-all-forms-of-malnutrition-from-evidence-to-programmes-and-policy

30. Promotion. OODPAH. Environmental Health [Internet]. Environmental Health , U.S. Department of Health & Human Services 2020. 2020. Available from: https://www.healthypeople.gov/2020/topics-objectives/topic/environmental-health

31. Promotion OODPAH. Oral Health, U.S. Department of Health & Human Services 2020 [Internet]. The U.S. Department of Health and Human Services. 2020. Available from: https://www.healthypeople.gov/2020/topics-objectives/topic/oral-health

32. World Health Organization. Pharmaceutical products [Internet]. World Health Organisation. 2020. Available from: https://www.who.int/topics/pharmaceutical_products/en/

33. WHO. Emergency care [Internet]. WHO. 2020. Available from: https://www.who.int/health-topics/emergency-care#tab=tab_1

34. Khattab E, Sabbagh A, Aljerian N, Binsalleeh H, Almulhim M, Alqahtani A, et al. Emergency medicine in Saudi Arabia: A century of progress and a bright vision for the future. Int J Emerg Med. 2019;12(1).
